# Nuclear m^6^A reader YTHDC1 regulates the scaffold function of LINE1 RNA in mouse ESCs and early embryos

**DOI:** 10.1007/s13238-021-00837-8

**Published:** 2021-04-22

**Authors:** Chuan Chen, Wenqiang Liu, Jiayin Guo, Yuanyuan Liu, Xuelian Liu, Jun Liu, Xiaoyang Dou, Rongrong Le, Yixin Huang, Chong Li, Lingyue Yang, Xiaochen Kou, Yanhong Zhao, You Wu, Jiayu Chen, Hong Wang, Bin Shen, Yawei Gao, Shaorong Gao

**Affiliations:** 1 Institute for Regenerative Medicine, Shanghai East Hospital, Shanghai Key Laboratory of Signaling and Disease Research, Frontier Science Center for Stem Cell Research, School of Life Sciences and Technology, Tongji University, Shanghai, 200120 China; 2 Clinical and Translation Research Center of Shanghai First Maternity & Infant Hospital, Shanghai Key Laboratory of Signaling and Disease Research, Frontier Science Center for Stem Cell Research, School of Life Sciences and Technology, Tongji University, Shanghai, 200092 China; 3 State Key Laboratory of Reproductive Medicine, Department of Prenatal Diagnosis, Women’s Hospital of Nanjing Medical University, Nanjing Maternity and Child Health Care Hospital, Nanjing Medical University, Nanjing, 211166 China; 4 School of Life Sciences, Peking University, Beijing, 100871 China; 5 Peking-Tsinghua Center for Life Sciences, Peking University, Beijing, 100871 China; 6 Department of Chemistry and Institute for Biophysical Dynamics, The University of Chicago, Chicago, IL 60637 USA; 7 Howard Hughes Medical Institute, Chicago, IL 60637 USA

**Keywords:** YTHDC1, LINE1-scaffold complex, 2-cell, retrotransposons, H3K9me3

## Abstract

N^6^-methyladenosine (m^6^A) on chromosome-associated regulatory RNAs (carRNAs), including repeat RNAs, plays important roles in tuning the chromatin state and transcription, but the intrinsic mechanism remains unclear. Here, we report that YTHDC1 plays indispensable roles in the self-renewal and differentiation potency of mouse embryonic stem cells (ESCs), which highly depends on the m^6^A-binding ability. *Ythdc1* is required for sufficient rRNA synthesis and repression of the 2-cell (2C) transcriptional program in ESCs, which recapitulates the transcriptome regulation by the LINE1 scaffold. Detailed analyses revealed that YTHDC1 recognizes m^6^A on LINE1 RNAs in the nucleus and regulates the formation of the LINE1-NCL partnership and the chromatin recruitment of KAP1. Moreover, the establishment of H3K9me3 on 2C-related retrotransposons is interrupted in *Ythdc1*-depleted ESCs and inner cell mass (ICM) cells, which consequently increases the transcriptional activities. Our study reveals a role of m^6^A in regulating the RNA scaffold, providing a new model for the RNA-chromatin cross-talk.

**Supplementary Information:**

The online version contains supplementary material available at 10.1007/s13238-021-00837-8.

## INTRODUCTION

In eukaryotes, N^6^-methyladenosine (m^6^A) is the most prevalent internal modification of messenger RNAs (mRNAs), repeat RNAs and long non-coding RNAs (lncRNAs) (Dominissini et al., 2012; Meyer et al., 2012; Chelmicki et al., 2021), and this mark is involved in the regulation of various RNA-related processes, including pre-mRNA splicing, nuclear RNA transport, mRNA translation and RNA decay (Wang et al., 2014; Wang et al., 2015; Xiao et al., 2016; Roundtree et al., 2017b; Shi et al., 2017). As a reversible mark, the m^6^A RNA methylation participates in the regulation of many essential biological events, including zygotic genome activation, cell fate transition and heat shock response (Batista et al., 2014; Chen et al., 2015; Zhou et al., 2015; Ivanova et al., 2017; Zhao et al., 2017). An interesting issue which requires further investigations is whether m^6^A plays a role in RNA-chromatin interactions.

The m^6^A-mediated regulation is dependent on the recognition of m^6^A-binding proteins, which are represented by the YTH domain family and HNRNP proteins (Roundtree et al., 2017a). For instance, YTHDF1-3, the cytoplasmic YTHDF proteins, were found to regulate the translation and decay of m^6^A-modified mRNA (Wang et al., 2014; Wang et al., 2015; Shi et al., 2017; Zaccara and Jaffrey, 2020). In nucleus, YTHDC1 is implicated in regulating the redistribution of m^6^A-marked transcripts from the nucleus to the cytoplasm (Roundtree et al., 2017b), pre-mRNA splicing (Xiao et al., 2016), co-transcriptional interplay (Li et al., 2020), the function of lncRNA *Xist* (Patil et al., 2016) and the decay of chromatin-associated RNAs (caRNAs) (Patil et al., 2016; Xiao et al., 2016; Li et al., 2020; Liu et al., 2020). Therefore, YTHDC1 may serve as a mediator for RNA-chromatin interactions to link different layers of epigenome. In mice, the nuclear binding of m^6^A by YTHDC1 was proved to be indispensable for gamete maturation and embryonic development (Kasowitz et al., 2018), suggesting that the YTHDC1-dependent regulation on nuclear RNAs is important for development processes.

To date, the diverse functions of caRNAs, including the functions of both coding and non-coding RNAs in gene regulation, have been revealed in different systems. In addition to the cis-acting regulation of nascent RNAs, a common trans-acting mode of nuclear RNAs is based on RNA-chromatin interactions; in this system, some RNAs act as a scaffold to recruit RNA-binding proteins (RBPs), and at least one component of the complex directly contacts with DNA-binding factors (Li and Fu, 2019). Recently, it was reported that RNAs transcribed from long interspersed nuclear element-1 (LINE1) form a LINE1-scaffold complex with NCL and KAP1 proteins, which regulates the exit from the 2-cell (2C)-like state in mouse ESCs (Percharde et al., 2018), and these findings provide new insights for investigating RNA-chromatin interactions.

In a recent study, we and collaborators found the existence of m^6^A marks on chromatin-associated regulatory RNAs (carRNAs), including LINE1 RNAs (Liu et al., 2020). The m^6^A modifications on carRNAs catalysed by METTL3 are further targeted by YTHDC1 for the RNA decay and alterations in the chromatin state. In addition to the METTL3-dependent processes, we found by surprise that YTHDC1 plays essential roles in the maintenance of ESCs, which is different from METTL3. We then identified METTL3-sensitive/insensitive m^6^A-marked LINE1 RNAs on chromatin, which were both targeted by YTHDC1 but appeared to be increased or stable upon *Ythdc1* depletion, respectively. Notably, the derepression of 2C-related transcripts, which could be caused by the dysfunction of the LINE1-scaffold complex, was also observed in *Ythdc1* deficient ESCs but not in *Mettl3* KO ESCs, suggesting the function of the LINE1 scaffold was related to YTHDC1 but not sensitive to METTL3. Further analyses revealed that YTHDC1 physically interacts with NCL and KAP1 to promote the formation of the LINE1-NCL-KAP1 complex. We then proved that YTHDC1 facilitates KAP1 recruitment to targets of the LINE1 scaffold, including 2C-related retrotransposons, and promotes the installation of histone 3 lysine 9 trimethylation (H3K9me3) as well as the transcription silencing on these sites in both ESCs and inner cell mass (ICM) cells, which further contributes to ESC identity and embryonic development.

## RESULTS

### Indispensable roles of *Ythdc1* in mouse ESCs

**Figure 1:**
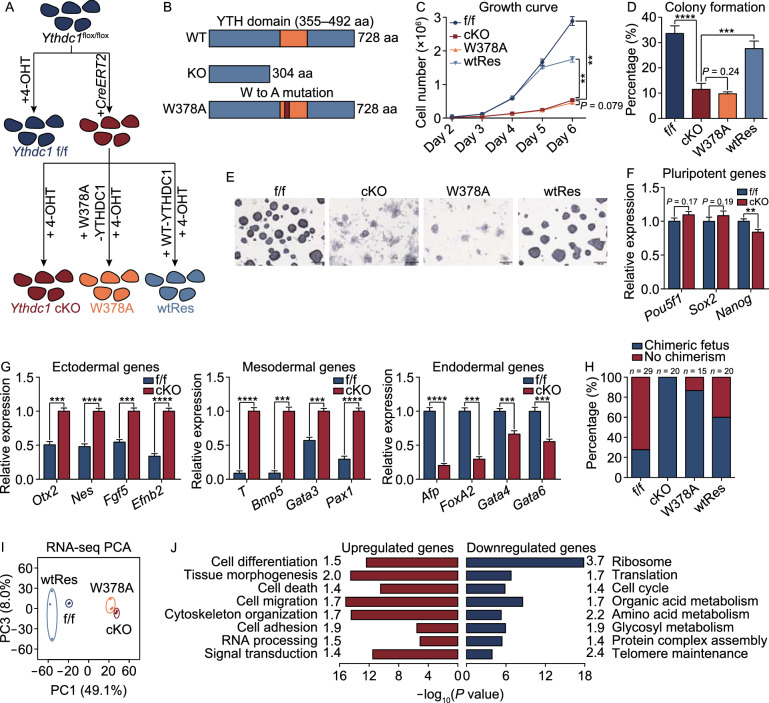
**YTHDC1 is essential for mouse ESCs**. (A) Strategy for functional studies of *Ythdc1* in ESCs. All cell lines were treated with 4-OHT for 3 days before harvest to ensure the depletion of endogenous *Ythdc1*. (B) Schematic of mouse wild-type (WT) YTHDC1, truncated YTHDC1 after the recombination (KO YTHDC1) and mutant YTHDC1 (W378A YTHDC1). aa, amino acid. (C) Growth curve showing that *Ythdc1* cKO and W378A ESCs exhibited a poor proliferation rate. Cell numbers on the last day were used to assess the significance. (D and E) Colony formation abilities of *Ythdc1* cKO and W378A ESCs were impaired revealed by AP staining. (F) RT-qPCR analysis showing the relative RNA level of key pluripotent markers in *Ythdc1* f/f and cKO ESCs. (G) RT-qPCR analysis showing that EBs derived from *Ythdc1* cKO ESCs exhibited the abnormal expression level of differentiation markers 7 days after *in vitro* differentiation. (H) *Ythdc1* cKO and W378A ESCs exhibited a weak ability to generate chimeric mice. (I) Principal component analysis (PCA) showing the transcriptome differences between each ESC line. (J) GO analysis of genes dysregulated in both *Ythdc1* cKO and W378A ESCs defined in Fig. S2E. Fold enrichment of each term is labeled in the plot. Data are presented as means with SDs (*n* = 3 in (C, F and G) and *n* = 4 in (D). Significance was calculated with unpaired two-tailed Student’s *t* test (**P < 0.01, ***P < 0.001, ****P < 0.0001). See also Figs. S1 and S2

To explore the functional mechanism of *Ythdc1* in mouse ESCs, we constructed a conditional knockout ESC line (*Ythdc1*^flox/flox^ with *CreERT2*, referred to as *Ythdc1* cKO), and the *Ythdc1*^flox/flox^ line (referred to as *Ythdc1* f/f) served as the control (See METHODS and Fig. S1A). Two rescue cell lines expressing WT YTHDC1 (referred to as wtRes) or m^6^A-binding site-mutated YTHDC1 (referred to as W378A) (Roundtree et al., 2017a; Liu et al., 2020) were also constructed to identify the roles of m^6^A (Fig. 1A and 1B). After treated with 4-hydroxytamoxifen (4-OHT), the endogenous *Ythdc1* in the cKO ESCs was almost depleted, and the YTHDC1 protein in the rescue cell lines was apparent but modestly less than that in *Ythdc1* f/f ESCs (Fig. S1B and S1C).

Consistent with the unsuccessful establishment of homozygous *Ythdc1*-depleted ESCs (Patil et al., 2016), we found that *Ythdc1* deficient ESCs could hardly support cell maintenance, as demonstrated by a markedly reduced proliferation rate and an impaired colony formation ability (Figs. 1C–E and S1D). An EdU incorporation assay showed that *Ythdc1* cKO ESCs failed to enter the S phase (Fig. S1E–F), and the apoptosis rate of *Ythdc1* f/f and cKO ESCs was comparable (Fig. S1G). However, despite *Ythdc1* depletion, key pluripotent markers appeared to be normal, with the exception of a slight decrease in the *Nanog* RNA and protein level (Figs. 1F and S1H). Notably, mutant YTHDC1 could hardly rescue these maintenance defects, and although the S phase proportion was not fully recovered, wtRes ESCs exhibited largely improved abilities for self-renewal and forming compacted colonies (Figs. 1C–E and S1D–F). We then evaluated the developmental potency of *Ythdc1* cKO ESCs through *in vitro* and *in vivo* differentiation. As shown, embryoid bodies (EBs) derived from *Ythdc1* cKO ESCs were markedly reduced with smaller sizes (Fig. S1I and S1J), and the markers of all three germ layers were expressed abnormally in *Ythdc1* cKO EBs (Fig. 1G). We then introduced a transgenic red fluorescence protein (RFP) into the four ESC lines and checked their integrating abilities in chimeric mice. Strikingly, no chimera was observed with the use of *Ythdc1* cKO ESCs, and W378A ESCs could hardly contribute to chimeric embryos either (Figs. 1H, S1K and S1L). These results suggest that YTHDC1 is essential for the self-renewal and proper differentiation of ESCs, and these roles are highly related to its m^6^A-recognition ability.

We then performed RNA-seq analysis of each line, and found that the transcriptome of *Ythdc1* cKO and W378A ESCs shared more similarities and was quite different from that of *Ythdc1* f/f or wtRes ESCs (Figs. 1I and S2A). Furthermore, the differentially expressed genes (DEGs) in *Ythdc1* cKO compared with f/f ESCs (546 upregulated and 1,373 downregulated genes, Fig. S2B and Data S1) could be largely corrected by WT YTHDC1 but not the W378A mutant (Fig. S2C and S2D). We then identified the transcriptome defects shared by *Ythdc1* cKO and W378A ESCs (Fig. S2E and Data S1) and found that the downregulated genes were closely related to ribosome- and translation-associated processes and that the upregulated genes were involved in cell differentiation and cell adhesion (Figs. 1J, S2F and S2G). These findings confirm that the regulation of YTHDC1 in ESCs is dependent on its m^6^A-recognition ability. Notably, the large-scale repression of ribosomal genes might lead to reduced rRNA synthesis, which was also observed in LINE1 knockdown (KD) ESCs and was considered responsible for the observed growth defects (Percharde et al., 2018).

### YTHDC1 recognizes differential m^6^A on LINE1 RNAs

**Figure 2:**
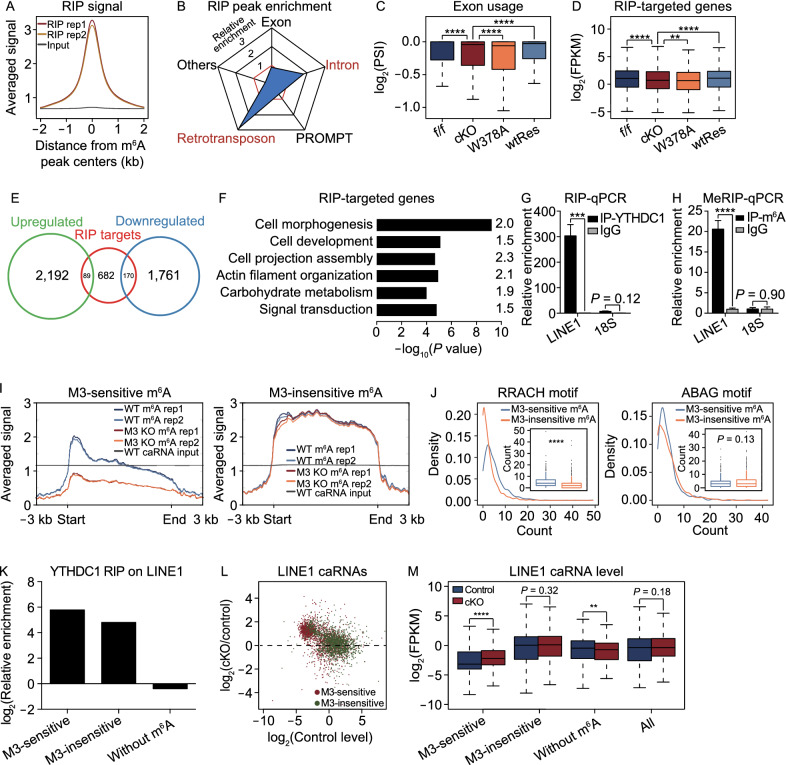
**Behaviors of YTHDC1-targeted RNAs in ESCs**. (A) YTHDC1 RIP-seq signal is enriched around the center of m^6^A peaks defined in the control ESCs. (B) YTHDC1 RIP peaks preferred to localize at introns and retrotransposons in genome in the control ESCs. PROMPT, promoter upstream transcript. (C) Usage of exons (calculated as PSI, see METHODS) adjacent to YTHDC1 RIP peaks was decreased upon *Ythdc1* depletion. (D) Expression level of YTHDC1-targeted coding genes is relatively lower in *Ythdc1* deficient ESCs. A total of 941 coding genes possessing RIP peaks (fold enrichment > 5) in their exons or introns were defined as YTHDC1 RIP targets. (E) Venn diagram showing the overlap of YTHDC1 RIP targets and dysregulated genes defined in Fig. S2E. (F) GO analysis of YTHDC1-targeted coding genes defined by RIP-seq in the control ESCs. Fold enrichment of each term is labeled in the plot. (G and H) YTHDC1 RIP-qPCR and m^6^A MeRIP-qPCR analysis show the preference of YTHDC1 binding and m^6^A deposition on nuclear LINE1 transcripts in the control ESCs, respectively. Relative enrichment was calculated as the percent of input relative to the negative control antibody IgG. Two independent reactions for each IP were performed. Data are presented as means with SDs (*n* = 3 technical replicates). Primers used in these assays target the consensus sequences of LINE1 ORF regions (Su et al., 2012). We have checked the m^6^A signal on 18S rRNA to ensure that 18S could be used as a negative control using the published nuclear RNA m^6^A MeRIP-seq data (Liu et al., 2020). (I) Profiles generated by deepTools showing the distribution of m^6^A IP signal on bodies of METTL3-sensitive m^6^A-marked (left) and METTL3-insensitive m^6^A-marked (right) LINE1 caRNAs in WT and *Mettl3* KO ESCs. M3, METTL3 or *Mettl3*. (J) Density plots and boxplots showing the occurrence frequency of RRACH (left) and ABAG (right) motifs in METTL3-sensitive /insensitive m^6^A peaks. M3, METTL3. (K) YTHDC1 RIP peaks were enriched on both METTL3-sensitive and METTL3-insensitive m^6^A-marked LINE1 transcripts in the nucleus. M3, METTL3. L1, LINE1. (L and M) METTL3-sensitive m^6^A-marked LINE1 RNAs increased their abundance on chromatin upon *Ythdc1* depletion, whereas METTL3-insensitive m^6^A-marked LINE1 caRNAs remained unchanged in *Ythdc1*-depleted ESCs. Scatter plot showing the relative level of m^6^A-marked LINE1 caRNAs in the control ESCs (x-axis) and fold change of the level upon *Ythdc1* depletion (y-axis) in L. A total of 1,989 METTL3-sensitive m^6^A-marked LINE1 transcripts, 1,664 METTL3-insensitive m^6^A-marked LINE1 transcripts (including 319 transcripts marked with both kinds of m^6^A) and 8,053 LINE1 transcripts without m^6^A sites were analyzed. M3, METTL3. caRNA m^6^A-seq data of WT and *Mettl3*^−/−^-1 ESCs (GSE133600) published by Liu et al. (2020) were used for the analyses in (I, J, K, L and M). Input data of caRNA m^6^A-seq in control and *Ythdc1* cKO ESCs were also downloaded from GSE133600 to quantify the relative abundance of LINE1 transcripts on chromatin in (L and M). Means of replicates were used to generate the summarized data in (C, D, L and M). Significance (**P < 0.01, ***P < 0.001, ****P < 0.0001) was calculated with two-tailed Student’s *t* test (paired in (C, D and M) and unpaired in (G, H and J)). See also Fig. S3

We subsequently questioned whether the transcriptional impact was related to the direct binding of YTHDC1. Through nuclear RNA-binding protein immunoprecipitation and sequencing (RIP-seq), we identified 12,237 peaks targeted by YTHDC1, and the RIP signal was enriched around the center of m^6^A peaks (Fig. 2A). We found that YTHDC1 binding was highly enriched in intronic regions and endogenous retrotransposons (Figs. 2B and S3A), which was consistent with the published regulatory roles of YTHDC1 on alternative splicing and repeat RNAs (Xiao et al., 2016; Liu et al., 2020). For coding genes, we found that most genes with YTHDC1 RIP peaks in exons also possessed peaks in introns (Fig. S3B), and a decreased usage of exons adjacent to YTHDC1 RIP peaks (Fig. 2C) and a slightly reduced expression level of YTHDC1-targeted genes was detected after *Ythdc1* depletion (Fig. 2D). However, most of YTHDC1-targeted genes were not included in the main transcriptome defects shared by *Ythdc1* cKO and W378A ESCs (Fig. 2E), and these genes were not enriched in ribosome- or translation-associated terms (Fig. 2F). These data indicate that most of the transcriptome dysregulation resulted from *Ythdc1* deficiency is not directly related to the co-transcriptional regulation of YTHDC1-targeted coding transcripts, a model proposed in a recently published study (Li et al., 2020).

We then investigated the binding of YTHDC1 on endogenous retrotransposons. We calculated the relative enrichment of YTHDC1 RIP peaks and caRNA m^6^A peaks (Liu et al., 2020) on major classes of retrotransposons. As shown, LINE1 possessed highly enriched YTHDC1 RIP peaks and m^6^A peaks (Fig. S3C and S3D), and these findings were further confirmed by RIP-qPCR and methylated RNA immunoprecipitation (MeRIP)-qPCR, respectively (Fig. 2G and 2H). In an earlier study, METTL3 was found to catalyze m^6^A methylation on carRNAs including LINE1 and facilitate RNA decay with YTHDC1 and the NEXT complex (Liu et al., 2020). However, the loss of *Mettl3* would not lead to a substantial loss in proliferation ability or prevent ESCs from forming compacted colonies (Batista et al., 2014; Geula et al., 2015), which is different from depletion of *Ythdc1* (Figs. 1C–E and S3E–H). These findings indicate that YTHDC1 might regulate some RNAs in a METTL3-independent manner, and we noticed that KD of LINE1 using antisense oligoes (ASO) also resulted in a dramatic decrease in ESC self-renewal with defects in entering S phase (Percharde et al., 2018), which highly indicates a functional defect of LINE1 upon *Ythdc1* depletion. We then defined m^6^A peaks absent in *Mettl3* KO ESCs as METTL3-sensitive peaks, and m^6^A peaks retained in *Mettl3* KO ESCs as METTL3-insensitive peaks using the published caRNA m^6^A-seq data of wild-type (WT) and *Mettl3* KO ESCs (Liu et al., 2020). Interestingly, METTL3-sensitive m^6^A peaks mainly located near the 5′ untranslated region (UTR) of LINE1 and decreased significantly upon *Mettl3* KO, whereas METTL3-insensitive m^6^A sites located throughout the LINE1 body, and did not change despite *Mettl3* KO (Fig. 2I). We next compared the consensus motifs of METTL3-sensitive/insensitive m^6^A peaks and found significant differences in the sequence patterns (Figs. 2J and S3I). The RRACH (R = G or A; H = A, C or U) motif, which is the consensus sequence recognized by METTL3, appeared more frequently in METTL3-sensitive m^6^A peaks compared to METTL3-insensitive m^6^A peaks. Meanwhile, a sequence pattern highly resembled the ACAGAGA motif, which is a conserved sequence on U6 snRNA recognized by METTL16 (Warda et al., 2017; Mendel et al., 2018), was found in METTL3-insensitive m^6^A peaks. These evidences suggest that METTL16 might be the potential methyltransferase which is responsible for the installing of METTL3-insensitive m^6^A on LINE1 caRNAs. We also compared the distribution of METTL3-sensitive/insensitive m^6^A peaks among LINE1 subfamilies, and found the abundance of L1Md_T was much higher in the METTL3-sensitive m^6^A marked LINE1 transcripts, but in general the top 5 families in the two groups were almost similar and were younger-aged families (Fig. S3J). Interestingly, although YTHDC1 binding was highly enriched on both METTL3-sensitive m^6^A-marked and METTL3-insensitive m^6^A-marked nuclear LINE1 RNAs (Figs. 2K and S3K), the relative level on chromatin and the response to *Ythdc1* depletion of these two groups of LINE1 RNAs was different revealed by the published caRNA-seq data (Liu et al., 2020). Generally, the amount of chromatin-occupied LINE1 RNAs with METTL3-insensitive m^6^A was relatively higher and not impacted by *Ythdc1* depletion (Fig. 2L and 2M). In contrast, the abundance of METTL3-sensitive m^6^A-marked LINE1 RNAs on chromatin significantly increased with the depletion of *Ythdc1* (Fig. 2M), which was consistent with the previous study (Liu et al., 2020). These results suggest that YTHDC1 may involve in the regulatory roles of LINE1 on chromatin in a METTL3-independent manner without impacting the chromatin occupancy of LINE1 RNAs. We also checked the level of LINE1 RNAs in total, and found the abundance of these two groups of LINE1 was relatively comparable, and both increased in response to *Ythdc1* depletion (Fig. S3L and S3M). Here LINE1 RNAs without m^6^A marks and the overall LINE1 RNAs served as negative controls. Although the unmarked LINE1 caRNAs slightly decreased upon *Ythdc1* depletion, the abundance of overall LINE1 was unchanged on chromatin and increased in total (Figs. 2L, 2M, S3L and S3M). These results suggest that YTHDC1 which binds to METTL3-inseneitive m^6^A may involve in the regulatory roles of LINE1 on chromatin. Different from reducing the occupancy of LINE1 RNAs on chromatin which was realized by LINE1 KD, *Ythdc1* depletion was more likely to impact the recruitment of epigenetic regulators in the LINE1-scaffold complex.

Taken together, we observed METTL3-insensitive m^6^A sites on LINE1 caRNAs which possessed differential location and motif preference compared with METTL3-sensitive m^6^A sites. Particularly, the binding of YTHDC1 to METTL3-insensitive m^6^A on LINE1 caRNAs might not participate in promoting the YTHDC1-triggered RNA decay, but might relate with the regulatory function of the LINE1-scaffold complex which was proved to be essential for the maintenance of mouse ESCs.

### *Ythdc1* depletion resembles the dysfunction of LINE1-scaffold complex

**Figure 3:**
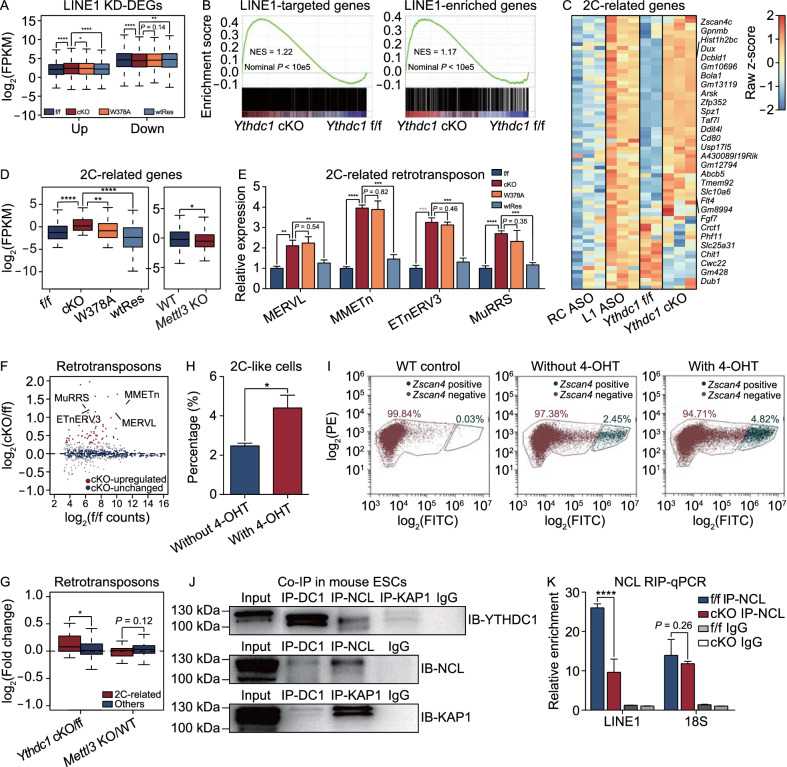
**YTHDC1 is involved in the function of LINE1-scaffold complex in ESCs**. (A) DEGs defined in LINE KD ESCs were similarly dysregulated in *Ythdc1* cKO and W378A ESCs. 498 LINE1 KD-upregulated genes and 248 LINE1 KD-downregulated genes with |log_2_(fold change)| > 0 and *P* value < 0.01 were defined by edgeR function in R using the published RNA-seq data (Percharde et al., 2018; GSE100939). Down, LINE1 KD-downregulated genes. Up, LINE1 KD-upregulated genes. (B) GSEA showing the global upregulation of LINE1 RNA-targeted genes (left, *n* = 2,397) and LINE1 sequence-enriched genes (right, *n* = 1,480) in *Ythdc1* cKO ESCs. These genes were defined by Lu et al. (2020). NES, normalized enrichment score. (C) Many 2C-related genes were consistently upregulated in LINE1 KD ESCs and *Ythdc1* cKO ESCs. A total of 76 genes with log_2_(fold change) > 1 (2C::tdTomato^+^ versus 2C::tdTomato^−^) were considered 2C-related genes based on the published RNA-seq data (Macfarlan et al., 2012). RC ASO, reverse complement of LINE1 ASO (served as a negative control). L1 ASO, LINE1 RNA KD by ASO. (D) 2C-related genes (Macfarlan et al., 2012; defined as in (C) were significantly upregulated in *Ythdc1* cKO ESCs but modestly downregulated in *Mettl3* KO ESCs. (E) RT-qPCR analysis showing that representative 2C-related retrotransposons were upregulated in *Ythdc1* cKO and W378A ESCs. (F) Scatter plot showing the relative abundance of retrotransposon subfamilies in *Ythdc1* f/f ESCs (x-axis) and the expression fold change upon *Ythdc1* depletion in ESCs (y-axis). 61 subfamilies with log_2_(fold change) > 0.3 and *P* value < 0.05 were defined as *Ythdc1* cKO-upregulated retrotransposons, and 304 subfamilies with |log_2_(fold change)| < 0.1 were defined as *Ythdc1* cKO-unchanged retrotransposons using edgeR function in R. Representative 2C-related retrotransposons consistently upregulated in LINE1 KD ESCs and *Ythdc1* cKO ESC are labeled in the plot. (G) 2C-related retrotransposons were upregulated upon *Ythdc1* depletion but not affected by *Mettl3* depletion in ESCs. A total of 46 2C-related retrotransposons were identified as retrotransposons with log_2_(fold change) > 1 (2C::tdTomato^+^ versus 2C::tdTomato^−^) based on the published study (Macfarlan et al., 2012), and other 565 repeat subfamilies serve as the control. (H and I) Proportion of 2C-like cells was significantly increased after *Ythdc1* depletion in the ESC population. Fluorescence signal plot showing GFP signal distribution (x-axis) in a representative *Ythdc1* cKO line with the *Zscan4c* promoter-GFP transgene in (I), and 2C-like cell population was determined according to the GFP signal in a WT control line without the transgene. (J) Co-IP in mouse ESCs showing that endogenous YTHDC1 interacted with both NCL and KAP1 proteins. DC1, YTHDC1. (K) NCL RIP-qPCR analysis showing that the association of NCL with nuclear LINE1 RNAs was attenuated in *Ythdc1* cKO ESCs. Relative enrichment was calculated as the percent of input relative to the negative control antibody IgG. Two independent reactions for each IP were performed. Data are presented as means with SDs in (E, H and K) (*n* = 3). Means of replicates were used to generate the summarized data in (A, D, F and G). Significance (*P < 0.05, **P < 0.01, ***P < 0.001, ****P < 0.0001) was calculated with two-tailed Student’s *t* test (paired in (A, D and H) and unpaired in (E, G and K)). See also Fig. S4

In mouse ESCs, LINE1 RNA acts as a nuclear scaffold for recruiting NCL/KAP1, repressing the 2C program, activating rRNA synthesis and promoting ESC self-renewal (Percharde et al., 2018). Considering the similar ribosome-associated and maintenance defects of *Ythdc1* cKO ESCs, we wondered whether the binding of YTHDC1 to METTL3-insensitive m^6^A on LINE1 caRNAs was related to the scaffold function of LINE1. We first defined DEGs in LINE1 ASO ESCs using the published RNA-seq data (Percharde et al., 2018) and found that many of these DEGs showed similar dysregulation in *Ythdc1* cKO and W378A ESCs, with a more significant increase in the level of LINE1 KD-upregulated genes (Figs. 3A and S4A). A recent study identified LINE1-targeted genes through chromatin isolation by RNA purification followed by sequencing (ChIRP-seq), and revealed that these genes as well as LINE1 sequence-enriched genes (referred to as LINE1-enriched genes) are repressed by the LINE1-scaffold complex (Lu et al., 2020). Notably, we found that *Ythdc1* deficiency in ESCs also led to similar upregulation of both LINE1-targeted and LINE1-enriched genes (Fig. 3B), which indicated that YTHDC1 might be important for the repressive role of LINE1. In addition, depletion of *Mettl3* would not induce the derepression of LINE1-targeted or LINE1-enriched genes (Fig. S4B), which further suggested that the YTHDC1-mediated regulatory function of LINE1 scaffold might not rely on METTL3.

As reported, the LINE1-NCL-KAP1 complex represses the 2C transcriptional program and promotes rRNA synthesis in mouse ESCs (Percharde et al., 2018). We then defined 2C-related genes and retrotransposons which were upregulated in 2C::tdTomato^+^ ESCs using the published data (Macfarlan et al., 2012), and observed consistent upregulation of 2C-related genes in LINE1 ASO and *Ythdc1* cKO ESCs (Figs. 3C, 3D and S4C). Consistently, we found many 2C-related retrotransposons such as MERVL and MMETn, were upregulated both in *Ythdc1* cKO and W378A ESCs (Figs. 3E–G, S4D and S4E). Remarkably, neither 2C-related genes nor retrotransposons were derepressed by the depletion of *Mettl3* in ESCs (Fig. 3D and 3G). These data further indicate that YTHDC1 and m^6^A is important for the regulation of 2C program by the LINE1-scaffold complex in ESCs, but this role may be independent of METTL3.

We next introduced a reporter of the 2C-like state, a *Zscan4* promoter-driven green fluorescence protein (GFP) (Rodriguez-Terrones et al., 2018), into *Ythdc1* cKO ESCs. Consistent with the upregulation of the 2C-related transcripts, the proportion of GFP-positive cells was significantly increased upon *Ythdc1* depletion (Fig. 3H and 3I), which indicated that the loss of *Ythdc1* promotes the transition of ESCs into the 2C-like state. Moreover, *Ythdc1* depletion also led to an obvious decrease in the rRNA level, and this decrease was not recovered in W378A ESCs (Fig. S4F). Taken together, these results indicate that *Ythdc1* deficiency recapitulates the dysregulation of 2C transcription program and rRNA synthesis caused by LINE1 KD, suggesting that the scaffold function of LINE1 is restrained by the depletion of *Ythdc1*.

### YTHDC1 facilitates the recruitment of NCL and KAP1 to LINE1-scaffold complex

In addition to the direct binding of YTHDC1 to LINE1 RNA, we wondered whether YTHDC1 could physically interact with the proteins in the LINE1-NCL-KAP1 complex. We firstly performed co-immunoprecipitation (Co-IP) with ESCs and found that YTHDC1 interacted with both NCL and KAP1 proteins (Fig. 3J), and the signal was conserved after the treatment of DNase or RNase (Fig. S4G). Then we expressed these mouse proteins in HEK293T cells with different combinations. Although the Co-IP signal seemed to be weaker, the interaction between mouse YTHDC1 and NCL proteins was observed when they were co-expressed in HEK293T cells (Fig. S4H). However, we found NCL might serve as a mediator as the interaction between KAP1 and YTHDC1 in HEK293T cells seemed to be stronger when NCL was also expressed (Fig. S4I).

**Figure 4:**
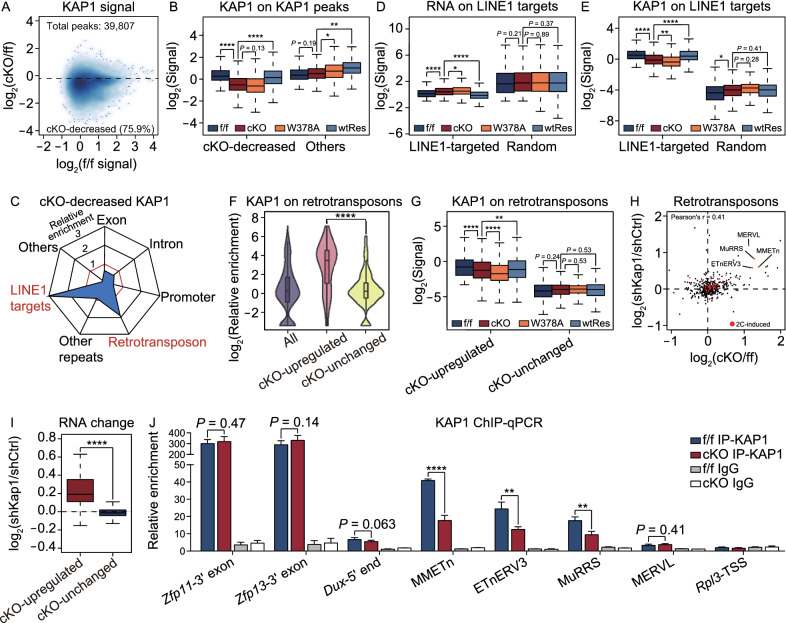
**YTHDC1 facilitates the genome recruitment of KAP1 in ESCs**. (A) Scatter plot showing the KAP1 ChIP signal in *Ythdc1* f/f ESCs (x-axis) and fold change of the signal upon *Ythdc1* depletion in ESCs (y-axis) on 39807 KAP1 peaks identified in f/f ESCs. A total of 30217 *Ythdc1* cKO-decreased KAP1 peaks were defined as KAP1 peaks with log_2_(fold change) < −0.2. (B) KAP1 ChIP signal was significantly decreased on *Ythdc1* cKO-decreased KAP1 peaks (defined in (A)) in *Ythdc1* cKO and W378A ESCs. (C) *Ythdc1* cKO-decreased KAP1 peaks (defined in (A)) were enriched at LINE1 RNA-targeted loci (Lu et al., 2020) and retrotransposons in genome. (D and E) RNA level was upregulated and KAP1 ChIP signal was decreased at LINE1 RNA-targeted loci (24202 regions defined by Lu et al., 2020) in *Ythdc1* cKO and W378A ESCs. An equal number of random loci serve as the control. (F) Violin plot showing that *Ythdc1* cKO-decreased KAP1 peaks (defined in (A)) were more enriched on *Ythdc1* cKO-upregulated retrotransposons compared to *Ythdc1* cKO-unchanged retrotransposons in ESCs. *Ythdc1* cKO-upregulated/unchanged retrotransposons were defined as in Fig. 3F. (G) KAP1 ChIP signal was significantly decreased on *Ythdc1* cKO-upregulated retrotransposons (defined as in Fig. 3F) in *Ythdc1* cKO and W378A ESCs. (H) Scatter plot showing fold change of the retrotransposon level upon *Ythdc1* depletion (x-axis) or *Kap1* KD (y-axis) in ESCs. Representative 2C-related retrotransposons consistently upregulated in *Ythdc1* cKO ESCs and *Kap1* KD ESCs are labeled in the plot. Pearson’s correlation coefficient was calculated in R. shCtrl, control shRNA expressed. shKap1, *Kap1* shRNA expressed. (I) *Ythdc1* cKO-upregulated retrotransposons were significantly more derepressed compared to *Ythdc1* cKO-unchanged retrotransposons upon *Kap1* KD in ESCs. *Ythdc1* cKO-upregulated/unchanged retrotransposons were defined as in Fig. 3F. (J) KAP1 ChIP-qPCR analysis showing the unaffected KAP1 binding at 5′ end of *Dux* locus, 3′ exons of *Zfp11*/*Zfp13* genes and MERVL elements, and the significantly decreased KAP1 binding at certain 2C-related retrotransposons upon *Ythdc1* depletion in ESCs. TSS of *Rpl3* gene which is not targeted by KAP1 serves as a negative control. Relative enrichment was calculated as the percent of input normalized to enrichment at the negative control intergenic chromosome 11 (int-chr11). Two independent reactions for each IP were performed. Data are presented as means with SDs (*n* = 3 technical replicates). Means of replicates were used to generate the summarized data in (A, B, D, E and G–I). Significance (*P < 0.05, **P < 0.01, ****P < 0.0001) was calculated with two-tailed Student’s* t* test (paired in (B, D, E and G) and unpaired in (F, I and J)). See also Fig. S5

Since the RNA or protein level of *Ncl* and *Kap1* was almost unchanged after *Ythdc1* depletion (Fig. S4J and S4K), we proposed that YTHDC1 might be important for the formation of the LINE1-NCL-KAP1 complex. To prove this hypothesis, we performed RIP-qPCR and found that the enrichment of NCL protein towards LINE1 RNA was significantly decreased in *Ythdc1* cKO ESCs, but NCL binding towards 18S rRNA was not impacted (Fig. 3K). We next performed chromatin immunoprecipitation followed by sequencing (ChIP-seq) to check the genome recruitment of KAP1. The ChIP signal intensity of KAP1 was highly reproducible between the two replicates for each ESC line (Fig. S5A), and our data on *Ythdc1* f/f ESCs were highly consistent with the published KAP1 ChIP-seq data on WT ESCs (De Iaco et al., 2017) (Fig. S5B and S5C). We then calculated the ChIP signal on KAP1 peaks identified in *Ythdc1* f/f ESCs. Notably, we observed 75.9% of KAP1 peaks appeared the loss of ChIP signal in *Ythdc1*-depleted ESCs which were defined as cKO-decreased KAP1 peaks, and the signal on the rest of peaks (referred to as “others” in the figures) was stable or increased upon *Ythdc1* depletion (Fig. 4A and 4B). We noticed that the cKO-decreased KAP1 peaks were highly enriched at LINE1 RNA-targeted loci (defined by Lu et al., 2020) and retrotransposons (Figs. 4C and S5D). Further analyses revealed that the transcription upregulation on LINE1-targeted loci was combined with the decreased KAP1 occupancy in *Ythdc1* cKO ESCs (Fig. S5E), which was also observed when the m^6^A recognition ability of YTHDC1 was disabled in W378A ESCs (Fig. 4D and 4E). Also, we found cKO-decreased KAP1 peaks were more significantly enriched at reactivated retrotransposons in *Ythdc1* cKO ESCs (defined as cKO-upregulated retrotransposons in Fig. 3F) compared to retrotransposons with stable abundance upon *Ythdc1* depletion (defined as cKO-unchanged retrotransposons; Figs. 4F and S5F), and as expected the binding of KAP1 on cKO-upregulated retrotransposons remarkably reduced in *Ythdc1* cKO and W378A ESCs (Fig. 4G). In addition, YTHDC1 RIP peaks did not show preference for cKO-upregulated retrotransposons (Fig. S5G), which excluded the possibility of potential impact from the direct binding of YTHDC1 to these retrotransposon RNAs. These results indicate that the transcriptional reactivation of LINE1-targeted genes and retrotransposons induced by *Ythdc1* deficiency might relate with the insufficient recruitment of KAP1 on corresponding loci. To test this relevance, we further established a *Kap1* KD ESC line, in which KAP1 binding was widely reduced on chromatin. Indeed, we observed the upregulation of LINE1-targeted and LINE1-enriched genes in *Kap1* KD ESCs (Fig. S5H) as well as the consistent reactivation of 2C-related genes and retrotransposons upon *Ythdc1* depletion or *Kap1* KD (Figs. 4H, 4I and S5I). Taken together, our data suggest that YTHDC1 might participate in the LINE1-mediated recruitment of KAP1 on LINE1-targeted loci and further regulate the transcription activities. In this model, the m^6^A marks on LINE1 caRNAs were essential for recruiting YTHDC1, and considering depletion of *Mettl3* could not induce the corresponding transcription derepression (Figs. 3D, 3G and S4B), the METTL3-insensitive m^6^A marks on LINE1 are more likely to be responsible for this YTHDC1 recruitment and therefore indispensable for the chromatin regulation of the LINE1-scaffold complex in ESCs.

Here we also noticed that compared to the global loss of KAP1 binding in the genome resulted from *Kap1* KD, the decrease of KAP1 occupancy caused by *Ythdc1* depletion was more specific. For instance, KAP1 binding towards the 3′ coding exons of certain ZFP genes, which were reported as canonical targets of KAP1 (Iyengar et al., 2011), remained relatively stable despite *Ythdc1* depletion (Figs. 4J and S5J). Meanwhile, the 5′ end of *Dux* gene, which is supposed to be targeted by KAP1 for the transcriptional regulation (De Iaco et al., 2017), appeared a slight but not significant decrease of KAP1 occupancy in *Ythdc1* cKO ESCs (Fig. 4J), although the reactivation of *Dux* was significant (Fig. S4C). Interestingly, although the transcriptional derepression of many 2C-related retrotransposons was observed in *Ythdc1* deficient ESCs (Fig. 3E and 3F), not all of them appeared significant loss of KAP1 binding. The MERVL elements, one of the well-known 2C-related retrotransposons, showed a low enrichment of KAP1 binding (Fig. S5K). This result is consistent with the previous study in which MERVL elements were reported to possess a low level of KAP1 enrichment at the flanks and internal regions, and were not supposed to be regulated by the KAP1/SETDB1-mediated deposition of H3K9me3 (Maksakova et al., 2013). In contrast, 2C-related retrotransposons including MMETn, ETnERV3 and MuRRS elements were enriched with cKO-decreased KAP1 peaks (Fig. S5F) and lost the KAP1 occupancy significantly in *Ythdc1* cKO ESCs (Figs. 4J and S5L). Therefore, the loss of KAP1 binding induced by the depletion of *Ythdc1* did not globally occur on the whole genome but preferred KAP1-targeted 2C-related retrotransposons, which were also among the targets of the LINE1-scaffold complex. In another word, the repression of 2C program in ESCs might be complicated, and some 2C-related retrotransposons were silenced by the YTHDC1 and LINE1-mediated KAP1 recruitment.

### YTHDC1 promotes H3K9me3 installation on 2C retrotransposons

**Figure 5:**
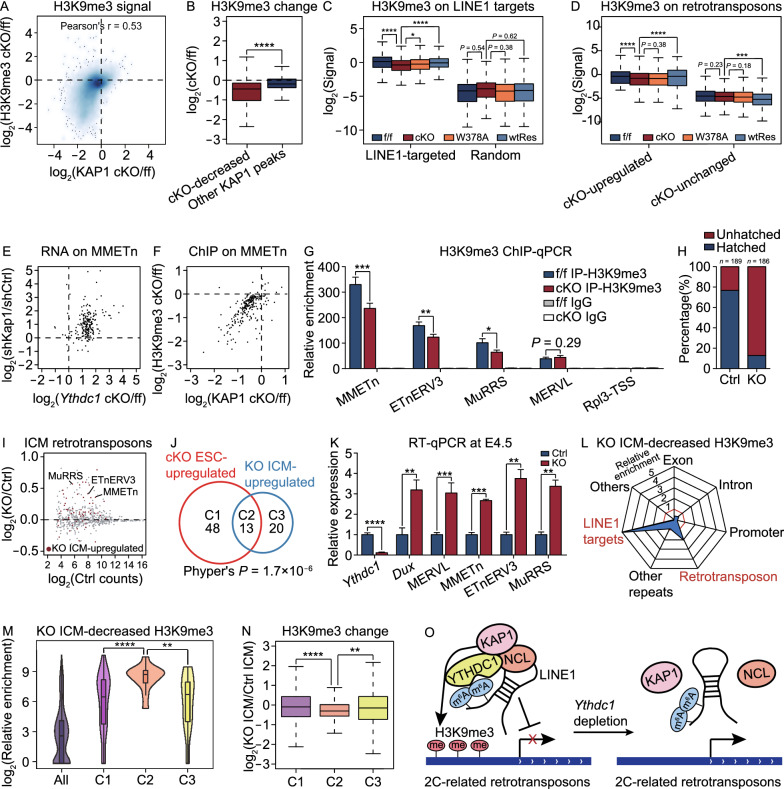
**YTHDC1 promotes H3K9me3 installation to regulate the repression of 2C retrotransposons in mouse ESCs and early embryos**. (A) Scatter plot showing fold change of KAP1 ChIP signal (x-axis) and H3K9me3 ChIP signal (y-axis) upon *Ythdc1* depletion in ESCs on KAP1 peaks identified in *Ythdc1* f/f ESCs. Pearson’s correlation was calculated in R. (B) H3K9me3 ChIP signal was more severely decreased on *Ythdc1* cKO-decreased KAP1 peaks (defined in Fig. 4A) upon *Ythdc1* depletion in ESCs. (C) H3K9me3 ChIP signal was significantly decreased on LINE1 RNA-targeted loci (24,202 regions defined by Lu et al., 2020) in *Ythdc1* cKO and W378A ESCs. An equal number of random loci serve as the control. (D) H3K9me3 ChIP signal was significantly decreased on *Ythdc1* cKO-upregulated retrotransposons (defined as in Fig. 3F) in *Ythdc1* cKO and W378A ESCs. (E) Scatter plot showing fold change of MMETn RNA level upon *Ythdc1* depletion (x-axis) or *Kap1* KD (y-axis) in ESCs. A total of 225 expressed MMETn elements are included in this plot. (F) Scatter plot showing fold change of KAP1 ChIP signal (x-axis) and H3K9me3 ChIP signal (y-axis) upon *Ythdc1* depletion in ESCs on genome loci of MMETn elements. A total of 225 expressed MMETn elements are included in this plot. (G) H3K9me3 ChIP-qPCR analysis showing the significantly decreased H3K9me3 level at certain 2C-related retrotransposons including MMETn, ETnERV3 and MuRRS elements and the unaffected H3K9me3 level at MERVL elements in *Ythdc1* cKO ESCs. TSS of *Rpl3* gene which lacks H3K9me3 marks serves as a negative control. Relative enrichment was calculated as the percent of input normalized to enrichment at the negative control intergenic chromosome 11 (int-chr11). Two independent reactions for each IP were performed. (H) Proportion of hatched blastocysts was decreased at E4.5 upon *Ythdc1* depletion in mouse embryos. Blastocysts with embryonic cells moving out of the zona pellucida were considered as having hatched. (I) Scatter plot showing the relative abundance of retrotransposon subfamilies in Ctrl ICM (x-axis) and fold change of the level upon *Ythdc1* depletion in ICM (y-axis). 33 subfamilies with log_2_(fold change) > 0.3 and *P* value < 0.05 were defined as retrotransposons upregulated in *Ythdc1* KO ICM using edgeR function in R. Representative 2C-related retrotransposons consistently upregulated in *Ythdc1* cKO ESCs and *Ythdc1* KO ICM are labeled in the plot. (J) Venn diagram showing the overlap of upregulated retrotransposons in *Ythdc1* cKO ESCs (defined as in Fig. 3F) and in *Ythdc1* KO ICM (defined as in (J)). Group C1, group C2 and group C3 retrotransposons are upregulated only in *Ythdc1* cKO ESCs, in both *Ythdc1* cKO ESCs and *Ythdc1* KO ICM, and only in *Ythdc1* KO ICM, respectively. Hypergeometric test was performed to calculate the significance of the overlap with phyper function in R. (K) RT-qPCR analysis showing that representative 2C-related retrotransposons were upregulated in E4.5 *Ythdc1* KO blastocysts. (L) *Ythdc1* KO ICM-decreased H3K9me3 peaks (defined in Fig. S6L) were enriched at LINE1 RNA-targeted loci (Lu et al., 2020) and retrotransposons. (M) Violin plot showing that *Ythdc1* KO ICM-decreased H3K9me3 peaks (defined in Fig. S6L) were more enriched at group C2 retrotransposons compared to group C1 and C2 retrotransposons. Group C1-C3 retrotransposons were defined in (J). (N) H3K9me3 ChIP signal was more severely decreased on group C2 retrotransposons compared to group C1 and C2 retrotransposons in *Ythdc1* KO ICM. Group C1-C3 retrotransposons were defined in (J). (O) Model showing the indispensable role of the m^6^A reader YTHDC1 in regulating the function of nuclear LINE1 scaffold in pluripotent cells. The LINE1 RNA scaffold is located at genomic loci of 2C-related retrotransposons and recognized by YTHDC1 through m^6^A modifications. YTHDC1 further promotes the recruitment of NCL-KAP1 and facilitates the deposition of H3K9me3, ensuring that these sites remain at a silent state and inhibiting the activation of 2C program. Data are presented as means with SDs in (G and K) (*n* = 3 technical replicates). Means of replicates were used to generate the summarized data in (A–F, I and N). Significance (*P < 0.05, **P < 0.01, ***P < 0.001, ****P < 0.0001) was calculated with two-tailed Student’s *t* test (paired in (C and D) and unpaired in (B, G, K, M and N)). See also Fig. S6

As a transcriptional corepressor, KAP1 is proposed to play a central role in repressing numerous endogenous retroviruses (ERVs) and recruit the H3K9 methyltransferase (KMTase) SETDB1 to class I and class II ERVs (Matsui et al., 2010; Maksakova et al., 2013) to reestablish H3K9me3 and transcriptional silence (Rowe et al., 2010). We therefore performed ChIP-seq to check the deposition of H3K9me3 in *Ythdc1* deficient ESCs (Fig. S6A). As expected, we observed a significant reduction in the H3K9me3 signal intensity on KAP1 peaks in *Ythdc1* cKO and W378A ESCs, which was highly correlated with the loss of KAP1 occupancy (Figs. 5A, 5B and S6B), especially at LINE1 RNA-targeted loci (Fig. 5C). Similarly, the cKO-upregulated retrotransposons exhibited significant decreases of the H3K9me3 level upon *Ythdc1* depletion (Figs. 5D and S6C), whereas cKO-unchanged retrotransposons overall had a lower level of H3K9me3 and did not appear the loss of this mark (Fig. 5D). Notably, W378A mutant protein could not recover the H3K9me3 loss on LINE1-targeted loci or cKO-upregulated retrotransposons (Figs. 5C, 5D and S6C), indicating that the promotion of YTHDC1 on H3K9me3 establishment on these sites was dependent on the m^6^A recognition ability.

We then took MMETn for instance, which is a subfamily of class II ERVs and is upregulated in 2C embryos (Macfarlan et al., 2012). We found that most MMETn elements were reactivated in both *Ythdc1* cKO ESCs and *Kap1* KD ESCs (Fig. 5E), and meanwhile the loss of KAP1 binding and H3K9me3 deposition was observed on MMETn loci upon *Ythdc1* depletion (Fig. 4J, 5F and 5G). Here we noticed remarkable differences between MMETn and the class III ERV MERVL. As shown, the H3K9me3 enrichment was relatively low on MERVL elements, and the H3K9me3 ChIP signal did not decrease on MERVL elements as they did on MMETn elements upon *Ythdc1* depletion (Fig. S6D and S6E), which was consistent with the behavior of KAP1 occupancy on these sites (Fig. S5K and S5L). In addition, the H3K9me3 level in total barely changed in *Ythdc1* cKO ESCs (Fig. S6F). Therefore, we speculate that YTHDC1 participates in the H3K9me3 installation on specific 2C-related retrotransposons targeted by the LINE1-NCL-KAP1 complex, promoting the transcription repression of these elements in ESCs.

### YTHDC1 is essential for the repression of 2C retrotransposons in mouse blastocysts

The scaffold function of LINE1 has also been reported in mouse embryos, and this role promotes the silencing of the 2C program and the developmental progression past the two-cell stage (Percharde et al., 2018). Here, we sought to determine whether YTHDC1 is also essential in mouse embryonic development. Indeed, we found that *Ythdc1* KO embryos usually died before embryonic day 6.5 (E6.5, Fig. S6G), which was consistent with the finding of a published study (Kasowitz et al., 2018). A high level of the YTHDC1 protein has been found in MII oocytes and pre-implantation embryos, and the maternal depletion of *Ythdc1* causes oocyte developmental arrest before maturation (Kasowitz et al., 2018). In addition, since the YTHDC1 protein encoded by dormant maternal mRNAs during oocyte maturation still remains in *Ythdc1* KO embryos obtained from intercrosses of *Ythdc1*^+/−^ mice, it is difficult to study the function of *Ythdc1* before the four-cell stage. To more easily obtain *Ythdc1* KO embryos, we injected four single-guide RNAs (sgRNAs) and Cas9 mRNA into zygotes to remove the whole exon 4 of *Ythdc1* (See METHODS), and embryos injected with only Cas9 mRNA were regarded as the control (Ctrl). We found that *Ythdc1* KO embryos could develop to the blastocyst stage at E3.5 (Fig. S6H) but failed to hatch out of the zona pellucida at E4.5 (Fig. 5H), indicating the impaired developmental ability of *Ythdc1* KO blastocysts which was consistent with the post-implantation lethality of *Ythdc1* KO mice. Notably, similar to the difference between YTHDC1 and METTL3 in regulating ESC maintenance, the developmental defects induced by *Mettl3* KO in mouse embryos were observed at a later stage (approximately at E7.5; Geula et al., 2015), suggesting that the regulatory roles of YTHDC1 in early embryos might be METTL3-independent but related with the LINE1-scaffold complex.

We next dissociated and collected ICM cells from E4.5 Ctrl and *Ythdc1* KO blastocysts for RNA-seq (Fig. S6I) and ultra-low-input native ChIP-seq for H3K9me3 (Fig. S6J). The elimination of *Ythdc1* was confirmed by the entire loss of RNA-seq read coverage at exon 4 of *Ythdc1* in the KO ICM (Fig. S6K). Consistent with the consequence of *Ythdc1* depletion in ESCs, a group of retrotransposons were upregulated in *Ythdc1* KO ICM revealed by our analyses on the transcriptome data (Fig. 5I). We then classified retrotransposons according to the change in their RNA abundance upon *Ythdc1* depletion in ESCs and embryos (Fig. 5J and Data S2). We defined 48 retrotransposons upregulated only in *Ythdc1* cKO ESCs (group C1), 13 retrotransposons upregulated both in *Ythdc1* cKO ESCs and *Ythdc1* KO ICM (group C2), and 20 retrotransposons upregulated only in *Ythdc1* KO ICM (group C3). Indeed, 2C-related retrotransposons including MMETn, ETnERV3 and MuRRS elements were defined as group C2. The reverse transcription (RT)-qPCR was performed to confirm the derepression of representative 2C genes and retrotransposons in E4.5 *Ythdc1* KO blastocysts (Fig. 5K), which had been observed in *Ythdc1* deficient ESCs (Figs. S4C and 3E). We then identified peaks losing H3K9me3 signal in *Ythdc1* KO ICM as KO ICM-decreased H3K9me3 peaks (Fig. S6L), and found they were highly enriched on LINE1 RNA-targeted sites and retrotransposons (Fig. 5L). Detailed analyses revealed that KO ICM-decreased H3K9me3 peaks showed a preference for group C2 retrotransposons compared with group C1 and C3 retrotransposons (Fig. 5M). Meanwhile, the loss of H3K9me3 signal on group C2 retrotransposons upon *Ythdc1* depletion in ICM was more significant than that on group C3 retrotransposons (Figs. 5N, S6M and S6N), indicating that the YTHDC1-mediated H3K9me3 installation plays an important role in the transcription repression of group C2 retrotransposons in both ESCs and early embryos. We further checked the detailed behaviors of MMETn, and similar to our observations in *Ythdc1* cKO ESCs (Fig. 5E–G), most MMETn elements were upregulated and simultaneously lost H3K9me3 occupancy in *Ythdc1* KO ICM (Fig. S6O). Taken together, our data indicate that YTHDC1 is essential for the establishment of H3K9me3 on specific 2C retrotransposons and regulates retrotransposon silencing during the developmental process of mouse blastocysts.

## DISCUSSION

In this study, we uncover the important roles of the nuclear m^6^A reader YTHDC1 in maintaining the self-renewal and repressing the 2C-like transcription program in mouse ESCs. Similar observations were reported recently (Liu et al., 2021). Our data further refine the working model in which YTHDC1 binds the m^6^A-marked LINE1 RNAs on chromatin and regulates the formation of the LINE1-NCL-KAP1 complex to establish H3K9me3 modifications on 2C-related retrotransposons and repress the 2C program, which ensures the appropriate transcriptome and developmental potency of ESCs and early embryos (Fig. 5P).

As a nuclear m^6^A reader, YTHDC1 is localized in the YT bodies, which contain focal sites of transcription and are found adjacent to nuclear speckles (Nayler et al., 2000), and pervious data revealed that YTHDC1 directly binds m^6^A-marked nascent RNAs, chromatin-associated RNAs and transcripts of retrotransposons (Xiao et al., 2016; Liu et al., 2020). Therefore, YTHDC1 is naturally capable of working as a connecting mediator for the communication between RNA modifications and the chromatin. The YTHDC1-mediated co-transcriptional regulation was raised in two recent studies, in one of which YTHDC1 was reported to recruit KDM3B to promote H3K9me2 demethylation on m^6^A-associated chromatin regions (Li et al., 2020), and in another study YTHDC1 was considered to stabilize METTL3 on chromatin through the METTL3-catalysed m^6^A modifications of RNAs to facilitate the integrity of IAP heterochromatin in mouse ESCs (Xu et al., 2021). In these two models, the function of YTHDC1 is highly related with METTL3-catalysed m^6^A marks and works in a manner of cis-regulation. However, unlike the depletion of *Ythdc1* which resembles the dysfunction of LINE1 scaffold, loss of *Mettl3* would not result in the derepression of 2C program or the substantially impaired self-renew ability of ESCs. This evidence suggests that the m^6^A modifications on the LINE1-scaffold caRNAs, which are also recognized by YTHDC1, may not be catalyzed by METTL3. Indeed, we identified a group of LINE1 caRNAs with METTL3-insensitive m^6^A sites, and the motif enrichment analysis suggested that METTL16, another m^6^A methyltransferase (Warda et al., 2017; Mendel et al., 2018), might be responsible for the deposition of these modifications. As a supporting evidence, we established ESC lines conditionally knocked out of *Mettl3*, *Mettl14* or *Mettl16*, and found that the binding of YTHDC1 on LINE1 RNAs was sensitive to the depletion of either METTL3-METTL14 or METTL16, and *Mettl16* depletion could even cause a more serious loss than *Mettl3* depletion (Fig. S6P). Therefore, it would be very important to further investigate how different methyltransferases set up unique m^6^A modifications on LINE1 caRNAs, and why YTHDC1 is able to recruit different RBP complexes such as NEXT complex and NCL-KAP1 to specific kinds of LINE1 caRNAs in ESCs.

Recently, the transcripts of LINE1 were found to play important roles in regulating the chromatin structure and transcription activities in early embryo and mouse ESCs (Jachowicz et al., 2017; Percharde et al., 2018; Lu et al., 2020), and these roles are independent of the retrotransposition capacity. However, the functions of repeat RNAs are complicated and would be controversial in case they were perceived as an aggregate class. In the previous study, LINE1 ASO was used to inhibit the function of LINE1 RNAs (Percharde et al., 2018), which lacked the specificity and caused a general loss of LINE1 RNAs on chromatin. In this system, both the cis- and trans-regulation of LINE1 was disrupted, which made it complicated and controversial to explore the accurate molecular mechanism. In our study, we compared the phenotypes and the transcriptome features of *Mettl3* KO, *Ythdc1* cKO and LINE1 ASO ESCs, and finally focused on the YTHDC1-involved trans-acting model of the LINE1-scaffold complex, in which YTHDC1 recognized the METTL3-insensitive m^6^A marks and facilitated the LINE1-KAP1-mediated H3K9me3 installation on specific 2C retrotransposons. However even in our system, we could not attribute all the transcriptional reactivation to the failure of LINE1-KAP1-mediated H3K9me3 installation, and for instance MERVL elements are not preferred by KAP1 or H3K9me3 occupancy. For rDNA, although the reduction in the level of rRNAs and ribosomal genes, which could be induced by LINE1 KD, was also observed in *Ythdc1* cKO ESCs, the KAP1 occupancy or H3K9me3 level was barely impacted by the depletion of *Ythdc1* (data not shown). Therefore, the regulation on MERVL and rDNA might work in an indirect way or at least not through the KAP1-mediated H3K9me3 installation. It would be another interesting issue which needs further investigations.

Compared to the cis-acting regulation, which occurs near the transcription sites on chromatin, the LINE1-scaffold complex facilitated by YTHDC1 is more likely to work in a trans-acting manner and involves in the complicated chromatin regulation at widespread regions. Our study provides numerous implications for the basic regulation through RNA m^6^A and YTHDC1 as well as the cross-talk between LINE1 RNA, chromatin modifications and transcription activities. It also reminds us the function of YTHDC1 in the *Xist*-mediated transcriptional repression, which relies on RBM15 and RBM15B-installed m^6^A marks in female mouse ESCs (Patil et al., 2016). The authors did not clarify but proposed that the PRC complex might participate in the gene repression, implicating the multitudinous impaction of the RNA-chromatin cross-talk mediated by YTHDC1. It would be interesting to further uncover diverse RNA-scaffold complexes formed by differential carRNAs and facilitated by YTHDC1 in different cells and developmental processes, and explore abundant tissue-specific mechanisms of RNA-chromatin regulations.

In summary, our findings further explore the cross-layer interaction model between RNA and chromatin and highlight the potential regulatory roles of RNA modifications on carRNAs in tuning transcription and the chromatin state during the developmental processes.

## MATERIALS AND METHODS

### Cell line generation and cell culture

The ESC clone for *Ythdc1* cKO (C57BL/6N-*Ythdc1*^tm1a(EUCOMM)Wtsi^) was purchased from EuMMCR and microinjected into mouse blastocysts. The resulting chimeric mice were crossed with flipper mice to excise the FRT flanked selection cassette. *Ythdc1*^flox/flox^ ESCs were derived from *Ythdc1*^flox/flox^ blastocysts. 2 × 10^5^ ESCs were transfected with 200 ng PB-CAG-CreERT2-P2A-puromycin and 100 ng PBase by electroporation using Lonza Nucleofector-4D. 24 h later, electroporated cells were treated with 1 μg/mL puromycin to screen for *Ythdc1*^flox/flox^ ESC clones stably expressing *CreERT2*. To generate rescue ESC lines, *Ythdc1*^flox/flox^; *CreERT2* cells were electroporated with PB-CAG-Ythdc1-P2A-blasticidin and PB-CAG-Ythdc1-W378A-P2A-blasticidin, respectively. 24 h after the electroporation, 1 μg/mL puromycin and 10 μg/mL blasticidin was added to generate stable *Ythdc1*^flox/flox^; *CreERT2*; WT-*Ythdc1* and *Ythdc1*^flox/flox^; *CreERT2*; W378A-*Ythdc1* ESC lines.

ESCs were cultured in ESC medium (DMEM-high glucose (Sigma-Aldrich), 15% FBS (HyClone), 1× Nucleosides (Merck Millipore), 1 mmol/L L-Glutamine (Merck Millipore), 1× Non Essential Amino Acids (Merck Millipore), 0.1 mmol/L 2-Mercaptoethanol (Gibco), 1000 U/mL LIF (Gibco)) supplemented with 3 mmol/L CHIR-99021 (Selleck) and 1 mmol/L PD0325901 (Selleck). To knock out the endogenous *Ythdc1* in *Ythdc1*^flox/flox^; *CreERT2* and rescue lines, 2.5 μmol/L 4-OHT (Sigma-Aldrich) was added into the culture medium which made the Cre recombinase enter the nuclei and trigger the deletion of exon 5–7 at *Ythdc1* locus. ESCs of each line were harvested for further investigations after 72-h treatment of 4-OHT. The *Mettl3* KO ESC line was provided by Jun Liu (Peking University), which was referred to as *Mettl3*^−/−^-1 in the published study (Liu et al., 2020).

### Generation of knockout mouse embryos and isolation of ICM

To ensure the effective deletion of *Ythdc1* in most embryos, four single guide RNAs (sgRNAs; Data S3) targeting exon 4 of *Ythdc1* gene were designed. The sequence for each sgRNA was cloned into the sgRNA expression vector pUC57 and *in vitro* transcription was then performed using MEGAshortscript T7 Transcription Kit (Invitrogen). The Cas9 expression construct pST1374-Cas9-N-NLS-Flag-linker was linearized and transcribed using mmol/LESSAGE mmol/LACHINE T7 ULTRA Transcription Kit (Invitrogen). B6D2F1 female mice (approximately 8-week-old) were superovulated and mated with B6D2F1 male mice before zygotes were collected from the oviducts. The harvested zygotes were microinjected with a mixture containing 100 ng/μL Cas9 mRNA and 50 ng/μL each sgRNA, and the embryos were then cultured in G-1 PLUS medium (Vitrolife). To test the knockout efficiency, we used 50 injected embryos as the PCR template and amplified genomic fragments spanning exon 4 of *Ythdc1* gene (primers are listed in Data S3). The PCR product was ligated to pClone007 vector and the resulting product was then transformed into *E. coli* bacteria. We observed large fragment deletions in all of 30 sequenced bacterial colonies, indicating *Ythdc1* was efficiently knocked out by the CRISPR/Cas9 system in most embryos.

For the isolation of ICM cells, E4.5 blastocysts were incubated in Ca^2+^-free Chatot-Ziomek-Bavister (CZB) medium for 30 min to disrupt the cell-cell junction. ICM cells were then distinguished and collected according to their sizes and shapes with the aid of a piezo-driven micromanipulator.

### Alkaline phosphatase staining

AP staining was performed using the BCIP/NBT Alkaline Phosphatase Color Development Kit (Beyotime). ESCs were fixed with 4% paraformaldehyde for 10 min at room temperature before they were washed with PBS twice and stained with the BCIP/NBT Solution following manufacturer’s protocol.

### Growth curve and colony formation assay

ESCs were dissociated by 0.05% trypsin-EDTA and the cell density was determined with a counting chamber. Cells were then plated in 12-well plates at 20,000 cells per well. 2 days, 3 days, 4 days, 5 days and 6 days after the passaging, cell number in each well was counted to plot the growth curve.

To analyze the ability of forming colonies, ESCs were digested and counted before they were seeded into 24-well plates at 500 cells per well. Three days later, cells were fixed and AP staining was performed as described above. AP^+^ colonies were then counted under an inverted microscope to calculate the percentages of cells which were able to form colonies in each line.

### Apoptosis assay and EdU incorporation

ESCs were digested and collected for the apoptosis assay, which was performed according to the manufacturer’s instructions of Annexin V-FITC Apoptosis Detection Kit (Beyotime). For the cell cycle analysis, 10 μmol/L EdU was added to the culture medium 2 h before the cells were harvested. The EdU labeling was then performed following the instructions of Click-iT Plus EdU Alexa Fluor 647 Flow Cytometry Assay Kit (Invitrogen). The percentages of apoptotic cells and S-phage cells were determined by fluorescence-activated cell sorting (FACS) and the results were analyzed through CytExpert software.

### Embryoid body differentiation

ESCs were trypsinized and resuspended at 50,000 cells/mL in LIF-free ESC medium. Hanging drops containing 20 μL of cell suspension were made on the lids of 10 cm-dishes and 10 mL of PBS was added in each dish below the drops. Two days later, the resulting EBs were transferred to non-adherent 6-well plates and cultured in LIF-free medium for another 5 days before they were harvested for gene expression detection.

### Generation of fluorescent protein-labeled ESC lines and chimeric mice

The pSicoR-Ef1a-mCherry-Puro plasmid together with the lentiviral packaging plasmids psPAX2 and pMD2.G were introduced into HEK293T cells using VigoFect reagent (Vigorous Biotechnology), and the transfected cells were cultured for 48 h to allow virus assembly and expansion. The supernatant medium containing viruses was mixed with 10% PEG 8000 (Sigma-Aldrich) and rotated for approximately 12 h at 4 °C. The mixture was then pelleted and resuspended with ESC medium, and the concentrate was subjected to infect ESCs of each line. After the infection, the RFP^+^ cells were screened and collected using FACS.

To generate chimeric mice, the RFP-labeled ESCs were trypsinized and approximately 10 ESCs were microinjected into each E3.5 blastocyst derived from the ICR mouse strain. These embryos were then implanted into the uteri of female pseudo pregnant ICR mice. 11 days after the implantation, the surrogate mothers were dissected and fetuses were harvested for further investigations. The extent of chimerism in each fetus was determined by the percentage of RFP^+^ cells using FACS analysis on the skin tissues.

### Detection of the 2C-like cell proportion within ESC culture

The sequence encoding a *Zscan4c* promoter-driven GFP were constructed into the Fugw plasmid, which was then introduced into *Ythdc1* cKO ESCs through lentivirus infection as described above. Cell lines with identical genetic materials were established from individual ES colonies after the infection, and three independent lines were used for subsequent analysis. To compare the 2C-like cell proportion before and after *Ythdc1* depletion, the three lines were cultured in ESC medium without or with 4-OHT for 3 days and subjected to FACS analysis. Cells with credible GFP expression were considered to be in a 2C-like state.

### Short hairpin RNA (shRNA)-mediated gene silencing

The sequence encoding each shRNA was constructed into the shRNA expression vector pSicoR-RFP, which was then introduced into WT ESCs through lentivirus infection as described above. The successfully infected ESCs were screened by FACS. shRNA sequences are listed in Data S3.

### Western blot

Cells were harvested and subjected to 1× Omni-Easy Protein Sample Loading Buffer (EpiZyme) for protein denaturing. Proteins were then separated by 10% SDS/PAGE and Western blot was performed by standard procedures. Primary antibodies were used as follows: anti-GAPDH (Proteintech 60004; 1:3,000), anti-YTHDC1 (Abcam ab122340; 1:1000), anti-NANOG (Abcam ab80892; 1:2,000), anti-KAP1 (Abcam ab22553; 1:2,000), anti-NCL (Proteintech 10556; 1:2,000), anti-H3 (Abcam ab1791; 1:3,000), anti-H3K9me3 (Abcam ab8898; 1:3,000). HRP-conjugated secondary antibodies (Santa Cruz; 1:5,000) were then applied. Protein signals were measured using High-sig ECL Western blot Substrate (Tanon) and visualized with ChemiDoc MP imaging system.

### Co-immunoprecipitation

Approximately 5 × 10^6^ cells were pelleted and sheared in lysis buffer (10 mmol/L HEPES, pH 7.4, 150 mmol/L NaCl, 2 mmol/L EDTA, 1% Nonidet P 40, 0.5 mmol/L DTT, protease inhibitor). The cell lysate was then incubated with specific antibody-bound Dynabeads Protein A/G (Invitrogen) in IP buffer (50 mmol/L HEPES, pH 7.4, 200 mmol/L NaCl, 2 mmol/L EDTA, 0.05% Nonidet P-40, 0.5 mmol/L DTT, protease inhibitor) for 8 h at 4 °C with rotation. Bound immunocomplexes were washed twice with IP buffer and 4 times with high-salt IP buffer (IP buffer with 500 mmol/L NaCl) before they were eluted by heat in 1× Omni-Easy Protein Sample Loading Buffer and subjected to immunoblotting.

### Reverse transcription and quantitative Real Time-PCR (RT-qPCR)

ESCs were disrupted in TRIzol Reagent (Invitrogen) and total RNAs were isolated by chloroform extraction coupled with isopropanol precipitation. RNAs of ICM cells were also isolated using TRIzol Reagent and chloroform, with the exception that 1/10 volume of 3 mol/L NaAc and 1 μL glycogen was added to the aqueous phase of each sample. RNAs were then precipitated by isopropanol and washed twice with 75% ethanol before they were eluted with nuclease-free water. cDNA was then synthesized using All-In-One RT MasterMix (Applied Biological Materials). qPCR was carried out using TB Green Premix Ex Taq II (Takara Bio) and monitored by 7500 Fast Real-Time PCR System, and three technical replicates were performed for each sample. Relative expression level of each gene was normalized to the reference gene *Hprt* for ESC samples or *H2afz* for embryo samples. qPCR primers for tested genes are listed in Data S3.

### Ribosomal RNA-free total RNA-Seq

Total RNAs were extracted as described above. For ESCs, two or three replicates for each sample were prepared, and 1 μg RNA per sample was subjected to rRNA elimination and RNA-seq library generation using VAHTS Total RNA-seq (H/M/R) Library Prep Kit (Vazyme) following manufacturer’s instructions. For ICM cells, two replicates for each sample were prepared, and purified RNAs were subjected to library generation using SMARTer Stranded Total RNA-Seq Kit (Takara Bio). Libraries were sequenced on the Illumina NovaSeq 6000 platform with paired ends and 150-bp read lengths at Berry Genomics.

### Nuclear RNA-binding protein immunoprecipitation

The RIP for YTHDC1 protein was performed according to manufacturer’s instructions of Nuclear RNA-Binding Protein Immunoprecipitation Kit (Merck Millipore). Briefly, two IP replicates were prepared, and 5 × 10^6^ WT ESCs per reaction were cross-linked in 0.3% formaldehyde for 10 min at room temperature and neutralized by 1× glycine. The fixed cells were pelleted by centrifugation and lysed with nuclei isolation buffer. The isolated nuclei were then pelleted and subjected to sonication using the Covaris M220 with conditions as follows: peak power 75, duty factor 20, cycles/burst 200, 5 min. 1/20 of the sonication product was saved as the input and the rest was incubated with YTHDC1 or NCL antibody-coated Dynabeads Protein A/G for 12 h with rotation at 4 °C. After the incubation, beads were washed and the immunoprecipitated complex was eluted as instructed. RNAs of the saved input and the IP product were extracted and subjected to either library generation using VAHTS Total RNA-seq (H/M/R) Library Prep Kit, or reverse transcription for qPCR. The paired-end sequencing was performed as described above. Primers for RIP-qPCR are listed in Data S3.

### Nuclear methylated RNA immunoprecipitation

Nuclei of ESCs were isolated according to the procedure in the published study (Liu et al., 2020). Briefly, 10^7^ ESCs were washed with 1 mmol/L EDTA in chilled DPBS and pelleted by centrifugation before they were subjected to cell membrane lysis buffer (10 mmol/L Tris-HCl, pH 7.5, 0.05% Nonidet P-40, 150 mmol/L NaCl) and incubated on ice for 5 min. 2.5 volumes of chilled sucrose cushion (24% sucrose in lysis buffer) were then added to the cell lysate and the mixture was centrifuged at 4 °C with 15,000 ×*g* for 10 min. The supernatant was discarded and the pellet was regarded as cell nuclei and subjected to RNA extraction using TRIzol Reagent as described above without denaturation. RNAs containing m^6^A modifications were then enriched using EpiMark N6-Methyladenosine Enrichment Kit (New England BioLabs) following manufacturer’s instructions.

### Chromatin immunoprecipitation

For KAP1 and H3K9me3 ChIP in ESCs, two replicates for each reaction were prepared, and 5 × 10^6^ cells per sample were fixed in 1% formaldehyde and the cross-link was stopped by 125 mmol/L glycine. The crosslinked cells were pelleted and washed twice with PBS before they were subjected to lysis buffer (50 mmol/L Tris-HCl, pH 8.0, 10 mmol/L EDTA, 1% SDS, protease inhibitor). The sonication was then performed with the following conditions: peak power 75, duty factor 20, cycles 200, 10 min. The product was cleared by centrifugation, and 1/100 of the supernatant was saved as the input. Antibodies for KAP1 or H3K9me3 were pre-bound to Dynabeads Protein A/G, and the sheared chromatin was incubated with the beads in RIPA buffer (10 mmol/L Tris-HCl, pH 7.5, 140 mmol/L NaCl, 1% Triton X-100, 0.1% SDS, 0.5 mmol/L EGTA, 1 mmol/L EDTA, protease inhibitor) for 12 h at 4 °C with rotation. After the immunoprecipitation, beads were washed twice with RIPA buffer, 4 times with high-salt RIPA buffer (RIPA buffer with 500 mmol/L NaCl) and once with TE buffer (10 mmol/L Tris-HCl, pH 8.0, 10 mmol/L EDTA). The immune complexes were then eluted in elution buffer (20 mmol/L Tris-HCl, pH 7.5, 50 mmol/L NaCl, 1% SDS, 5 mmol/L EDTA, 0.2 mg/mL protease K) at 68 °C with shaking for 2 h. For DNA purification, the input and IP samples were mixed with an equal volume of DNA Extraction Reagent (Solarbio), and 1/10 volume of 3 mol/L NaAc together with 1 μL glycogen was added to the aqueous phase of each sample before DNAs were precipitated by isopropanol. Sequencing libraries from the purified DNAs were generated using HyperPlus Library Preparation Kit (Kapa Biosystem). Primers for ChIP-qPCR are listed in Data S3.

The ultra-low-input micrococcal nuclease-based native chromatin immunoprecipitation (ULI-NChIP) was performed as previously described (Brind’Amour et al., 2015; Liu et al., 2016) to capture the H3K9me3 status in ICM cells. Briefly, two replicates for each reaction were prepared, and the harvested cells were subjected to nuclei extraction buffer (10 mmol/L Tris-HCl, pH 8.0, 140 mmol/L NaCl, 5 mmol/L MgCl_2_, 0.6% Nonidet P-40, 1 mmol/L PMSF, protease inhibitor). The MNase Master mix (0.6 U/μL MNase (New England BioLabs), 1× MNase master buffer, 2 mmol/L DTT, 5% PEG 6000) was then added and the mixture was incubated at 25 °C for 10 min to allow MNase digestion. The reaction was stopped by 10 mmol/L EDTA, and 0.1% Triton X-100 together with 0.1% DOC was added to lyse the nuclear membrane. The released chromatin was then diluted with ChIP buffer (10 mmol/L Tris-HCl, pH 8.0, 90 mmol/L NaCl, 2 mmol/L EDTA, 0.1% Triton X-100, 0.1% DOC, 0.1 mmol/L PMSF, protease inhibitor). After 1/20 of the reaction was saved as the input, the rest was incubated with H3K9me3 antibody-coated Dynabeads Protein A/G for 12 h at 4 °C with rotation. The IP samples were washed twice with low-salt wash buffer (20 mmol/L Tris-HCl, pH 8.0, 150 mmol/L NaCl, 2 mmol/L EDTA, 1% Triton X-100, 0.1% SDS, protease inhibitor) and twice with high-salt wash buffer (wash buffer with 500 mmol/L NaCl). The washed beads were then incubated in hot elution buffer (100 mmol/L NaHCO_3_, 1% SDS) for 2 h at 65 °C with shaking. The eluted DNAs were further purified and subjected to sequencing library generation as described above. Paired-end 150-bp sequencing was also performed on ChIP libraries.

### RNA-Seq data processing and analysis

Sequencing reads were trimmed by Trim Galore (v 0.6.4) to remove adapter sequences with parameters: –phred33, –illumina, –clip_R1 9, –clip_R2 9, –paired. Reads were then aligned to Mouse genome version mm9 by the unique mapping of Tophat2 (v 2.1.1) (Trapnell et al., 2009), which reported the alignment with the best alignment score if multiple alignments were mapped for a given read. Expression level of individual genes in each sample was quantified by fragments per kilobase of exon model per million reads mapped (FPKM) using Cufflinks (v 2.2.1) (Trapnell et al., 2010), and genes with a low expression level (averaged FPKM < 0.1) were ignored. Differentially expressed genes were defined as genes with fold change > 2 using the cuffdiff function of Cufflinks. Gene ontology (GO) analysis for functional annotation of gene sets was performed on the DAVID website, which provided fold enrichment of each term and p values showing the significance, and clusters were summarized to several representative terms. Usage of individual exons in each sample was calculated as percent spliced in index (PSI), which was the ratio between reads including or excluding the designated exons (Schafer et al., 2015). caRNA expression data of control and *Ythdc1* cKO ESCs were downloaded from the GEO database under the accession number GSE133600 (Liu et al., 2020), and data were processed as described above. To quantify the relative level of each LINE1 transcript, we generated GTF files containing METTL3-sensitive m^6^A-marked LINE1 transcripts or METTL3-insensitive m^6^A-marked LINE1 transcripts, and used the cuffdiff algorithm to produce FPKM for individual transcripts. Gene set enrichment analysis (GSEA) was performed in the software (v 4.0.1) (Subramanian et al., 2005), which provided normalized enrichment scores and nominal p-values. To quantify RNA level for different kinds of retrotransposons, genomic regions of each repeat subfamily were defined and regions overlapped with coding genes were excluded; the aligned files were normalized according to the sequencing depth; the relative expression level of a subfamily was calculated based on the number of reads falling on its transcripts using the intersect function of BEDTools (v 2.20.1) (Quinlan, 2014). To compare the read coverage at specific sites between samples, the normalized BAM files were converted to BedGraph files using the genomecov function of BEDTools, which were further converted to BigWig files using bedGraphToBigWig (Kent et al., 2010) and visualized in Integrative Genomics Viewer (IGV) (Thorvaldsdottir et al., 2013). The normalized BigWig files were also used to quantify RNA signal on designated regions with bigWigSummary.

### m^6^A-seq, RIP-Seq and ChIP-Seq data processing and analysis

caRNA m^6^A-seq data of WT and *Mettl3*^−/−^-1 ESCs were downloaded from the GEO database under the accession number GSE133600 (Liu et al., 2020), and reads were similarly trimmed and uniquely mapped to the reference genome mm9 using Tophat2. The aligned reads were then split to strand-specific BAM files using SAMtools (v 1.3) (Li et al., 2009) and m^6^A peaks were defined using the callpeak function of MACS2 (v 2.1.1) (Zhang et al., 2008) with the following parameters: -g 1.3e8, –BDG, –SPMR, –QVALUE 0.01, –tsize 150, –nomodel, –extsize 150. To calculate the relative enrichment of m^6^A peaks on different retrotransposons, the percentage of peaks falling on each subfamily was divided by the percentage of input reads falling on corresponding regions. Peaks detected in WT samples but absent in *Mettl3* KO samples were defined as METTL3-sensitive m^6^A peaks, and peaks defined in both WT and *Mettl3* KO samples were defined as METTL3-insensitive m^6^A peaks. LINE1 transcripts containing different kinds of m^6^A marks were then defined using the intersect function of BEDTools with annotation files for retrotransposons downloaded from UCSC Table Browser. Signal distribution profiles were generated using deepTools (v 2.3) (Ramírez et al., 2016). Motif enrichment analysis was performed using the findMotifsGenome function of HOMER (v 4.11) (Heinz et al., 2010) with parameters: -rna, -len 6.

Raw reads from RIP-seq libraries were trimmed and uniquely mapped to the reference genome mm9 using Tophat2 as described above. The output BAM files were used to define RIP peaks by MACS2 with the following parameters: -g mm, –BDG, –SPMR, –QVALUE 0.01. The intersected peaks defined in both of the replicates were considered as high confidence peaks. To compare the enrichment of RIP peaks on different elements, genomic regions of promoter upstream transcripts (PROMPTs, 2 kb upstream of the transcription start site), exons, introns and retrotransposons were defined; the percentage of peaks falling on each element was then calculated, which is further divided by the percentage of input reads falling on the corresponding element, and this ratio is regarded as the relative enrichment for the element. The relative enrichment of RIP on individual repeat subfamilies was calculated based on similar methods.

ChIP-seq reads were trimmed and uniquely mapped to mm9 using Bowtie2 (v 2.2.9) (Langmead and Salzberg, 2012). The output SAM files were converted to sort BAM files using SAMtools, and duplicate reads were removed using the MarkDuplicates tool of Picard (v 2.22.2). The filtered files were further normalized according to the sequencing depth. Peak calling was performed using MACS2 and the relative peak enrichment was computed as described above. The BedGraph files generated by MACS2 were further converted to BigWig files using bedGraphToBigWig (v 4) (Kent et al., 2010). Signal tracks were also visualized in IGV. The normalized BigWig files of ChIP and input samples were used to quantify the averaged signal at designated regions by bigWigSummary, and the ratio (IP/input) was regarded as ChIP signal intensity.

### Statistical analysis

All statistical analyses were performed in R (v 3.5.1). Numbers of replicates in each test are labeled in the graph or stated in the legend, and the error is reported in the graph as the standard deviation (SD). The statistical significance (*P < 0.05, **P < 0.01, ***P < 0.001, ****P < 0.0001) between groups was determined by two-tailed Student’s *t* test. For sequencing data, the mean of replicates are presented in the figures including the boxplots and scatter plots.

## Supplementary Information

Below is the link to the electronic supplementary material.

Supplementary material 1 (XLSX 1042 kb)

Supplementary material 2 (XLSX 75 kb)

Supplementary material 3 (XLSX 12 kb)

Supplementary material 4 (PDF 3012 kb)
