# Correction to: Nuclear m6A reader YTHDC1 regulates the scaffold function of LINE1 RNA in mouse ESCs and early embryos

**DOI:** 10.1007/s13238-021-00853-8

**Published:** 2021-08-25

**Authors:** Chuan Chen, Wenqiang Liu, Jiayin Guo, Yuanyuan Liu, Xuelian Liu, Jun Liu, Xiaoyang Dou, Rongrong Le, Yixin Huang, Chong Li, Lingyue Yang, Xiaochen Kou, Yanhong Zhao, You Wu, Jiayu Chen, Hong Wang, Bin Shen, Yawei Gao, Shaorong Gao

**Affiliations:** 1 Institute for Regenerative Medicine, Shanghai East Hospital, Shanghai Key Laboratory of Signaling and Disease Research, Frontier Science Center for Stem Cell Research, School of Life Sciences and Technology, Tongji University, Shanghai, 200120 China; 2 Clinical and Translation Research Center of Shanghai First Maternity & Infant Hospital, Shanghai Key Laboratory of Signaling and Disease Research, Frontier Science Center for Stem Cell Research, School of Life Sciences and Technology, Tongji University, Shanghai, 200092 China; 3 State Key Laboratory of Reproductive Medicine, Department of Prenatal Diagnosis, Women’s Hospital of Nanjing Medical University, Nanjing Maternity and Child Health Care Hospital, Nanjing Medical University, Nanjing, 211166 China; 4 School of Life Sciences, Peking University, Beijing, 100871 China; 5 Peking-Tsinghua Center for Life Sciences, Peking University, Beijing, 100871 China; 6 Department of Chemistry and Institute for Biophysical Dynamics, The University of Chicago, Chicago, IL 60637 USA; 7 Howard Hughes Medical Institute, Chicago, IL 60637 USA

## Correction to: Protein Cell (2021) 10.1007/s13238-021-00837-8

In the original publication of the article figure 1 is incorrectly published. The correct Figure 1 is provided in this correction.

**Figure 1:**
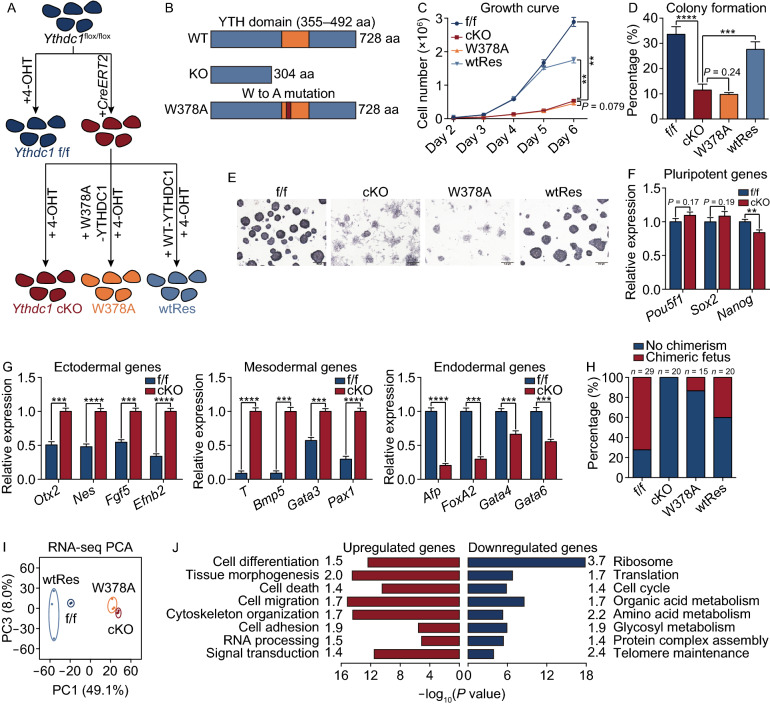
**YTHDC1 is essential for mouse ESCs**. (A) Strategy for functional studies of *Ythdc1* in ESCs. All cell lines were treated with 4-OHT for 3 days before harvest to ensure the depletion of endogenous *Ythdc1*. (B) Schematic of mouse wild-type (WT) YTHDC1, truncated YTHDC1 after the recombination (KO YTHDC1) and mutant YTHDC1 (W378A YTHDC1). aa, amino acid. (C) Growth curve showing that *Ythdc1* cKO and W378A ESCs exhibited a poor proliferation rate. Cell numbers on the last day were used to assess the significance. (D and E) Colony formation abilities of *Ythdc1* cKO and W378A ESCs were impaired revealed by AP staining. (F) RT-qPCR analysis showing the relative RNA level of key pluripotent markers in *Ythdc1* f/f and cKO ESCs. (G) RT-qPCR analysis showing that EBs derived from *Ythdc1* cKO ESCs exhibited the abnormal expression level of differentiation markers 7 days after *in vitro* differentiation. (H) *Ythdc1* cKO and W378A ESCs exhibited a weak ability to generate chimeric mice. (I) Principal component analysis (PCA) showing the transcriptome differences between each ESC line. (J) GO analysis of genes dysregulated in both *Ythdc1* cKO and W378A ESCs defined in Fig. S2E. Fold enrichment of each term is labeled in the plot. Data are presented as means with SDs (*n* = 3 in (C, F and G) and *n* = 4 in (D). Significance was calculated with unpaired two-tailed Student’s *t* test (**P < 0.01, ***P < 0.001, ****P < 0.0001). See also Figs. S1 and S2

