# Supplementary material for: Nuclear m6A reader YTHDC1 regulates the scaffold function of LINE1 RNA in mouse ESCs and early embryos
Source: Protein Cell. 2021 Apr 22;12(6):455–74. doi: 10.1007/s13238-021-00837-8 (PMC8160034; doi:10.1007/s13238-021-00837-8)
Supplement: Supplementary file 4 — Supplementary material 4 (PDF 3012 kb) [file 13238_2021_837_MOESM4_ESM.pdf]

Supplementary Figures for

Nuclear m^{6}A reader YTHDC1 regulates the scaffold function of LINE1 RNA in mouse ESCs and early embryos

Chuan Chen[1*], Wenqiang Liu[2*], Jiayin Guo[3], Yuanyuan Liu[3], Xuelian Liu[1], Jun Liu[4,5], Xiaoyang Dou[6,7], Rongrong Le[1], Yixin Huang[1], Chong Li[2], Lingyue Yang[2], Xiaochen Kou[1], Yanhong Zhao[1], You Wu[1], Jiayu Chen[2], Hong Wang[2], Bin Shen[3,#], Yawei Gao[1,#] and Shaorong Gao[1,2,8#]

Figure S1

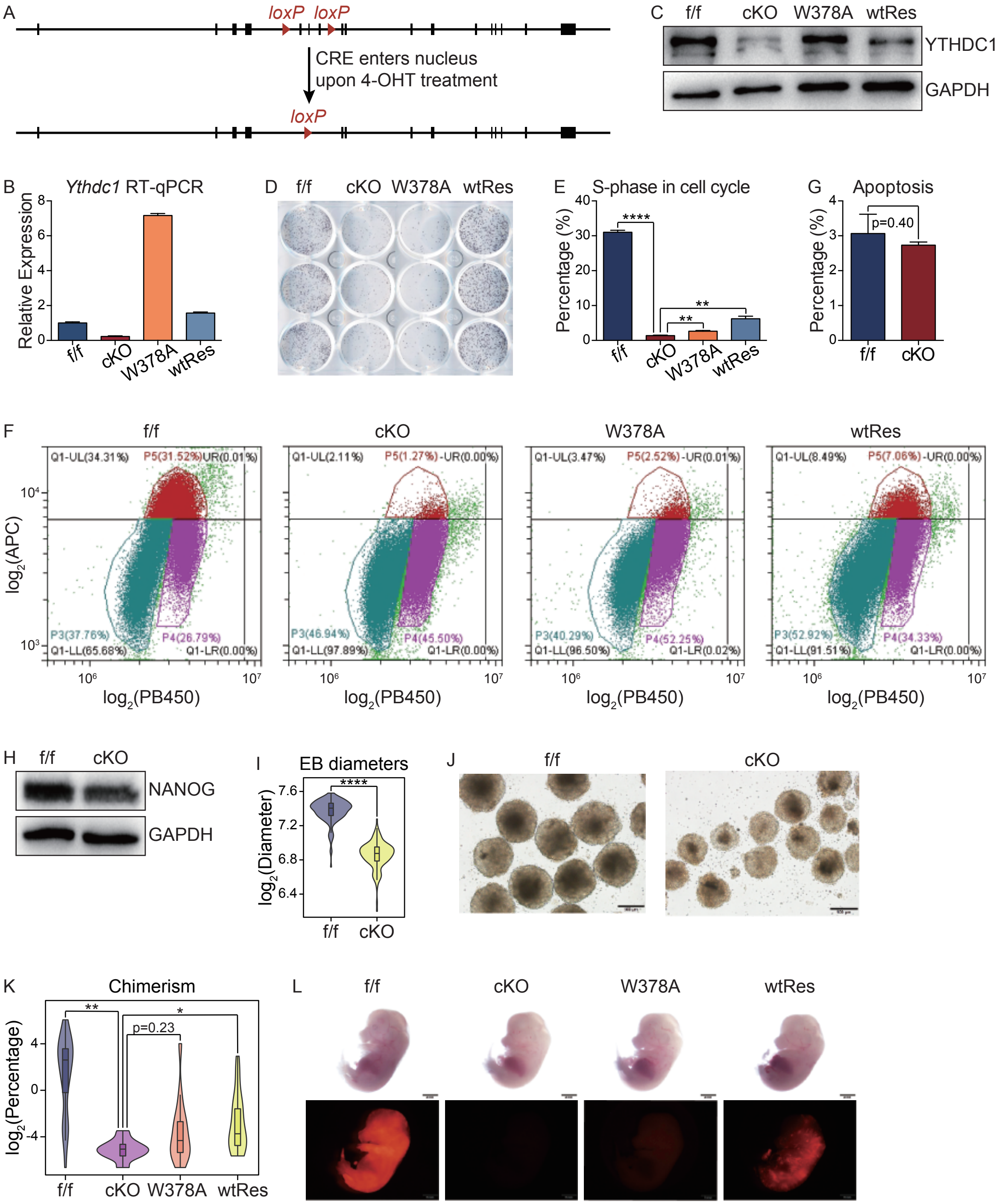
A
loxP loxP
CRE enters nucleus upon 4-OHT treatment
loxP
C f/f cKO W378A wtRes
YTHDC1
GAPDH
B Ythdc1 RT-qPCR
Relative Expression
f/f cKO W378A wtRes
D f/f cKO W378A wtRes
E S-phase in cell cycle
Percentage (%)
f/f cKO W378A wtRes
G Apoptosis
p=0.40
Percentage (%)
f/f cKO
F f/f cKO W378A wtRes
log2(APC)
log2(PB450)
H f/f cKO
NANOG
GAPDH
I EB diameters
log2(Diameter)
f/f cKO
J f/f cKO
K Chimerism
p=0.23
log2(Percentage)
f/f cKO W378A wtRes
L f/f cKO W378A wtRes

Fig. S1. Growth and Differentiation Defects Caused by *Ythdc1* Deficiency in ESCs, Related to Fig. 1.

(A) Strategy for *Ythdc1* conditional knockout in ESCs. Upon 4-OHT treatment, Cre recombinase enters the nuclei and triggers the deletion of exons 5-7 at endogenous *Ythdc1* locus in the cKO and rescue ESC lines.

(B and C) RT-qPCR analysis and western blotting showing that most of *Ythdc1* RNA and protein is eliminated in the cKO ESCs, and the ectopic expression of mutant or WT *Ythdc1* is sufficient in the rescue lines.

(D) AP staining showing that *Ythdc1* cKO and W378A ESCs exhibited the impaired colony formation ability (related to Fig. 1 D and 1 E).

(E and F) *Ythdc1* cKO and W378A ESCs could hardly enter the S phase revealed by the EdU incorporation assay. DNA content (PB450, x-axis) and EdU incorporation level (APC, y-axis) is shown in (F).

(G) Proportion of early apoptotic cells (defined as Annexin V^+ cells) was comparable in *Ythdc1* f/f and cKO ESCs.

(H) Western blotting showing that NANOG protein was slightly reduced in *Ythdc1* cKO ESCs.

(I and J) Violin plot showing that the diameters of embryoid bodies formed by *Ythdc1* cKO ESCs were significantly smaller than those formed by f/f ESCs 4 days after *in vitro* differentiation. Violin plot shows diameters of 132 EBs for each line in (I).

(K and L) *Ythdc1* cKO and W378A ESCs could hardly contribute to chimeric embryos (related to Fig. 1H). Violin plot shows the percentage of RFP^+ cells in E14.5 fetuses (n=20) derived from the injected blastocysts in (K).

Data are presented as means with SDs in (B, E and G) (n = 3). Significance was calculated with unpaired two-tailed Student's t test (* $p < 0.05$, ** $p < 0.01$, **** $p < 0.0001$).

Figure S2

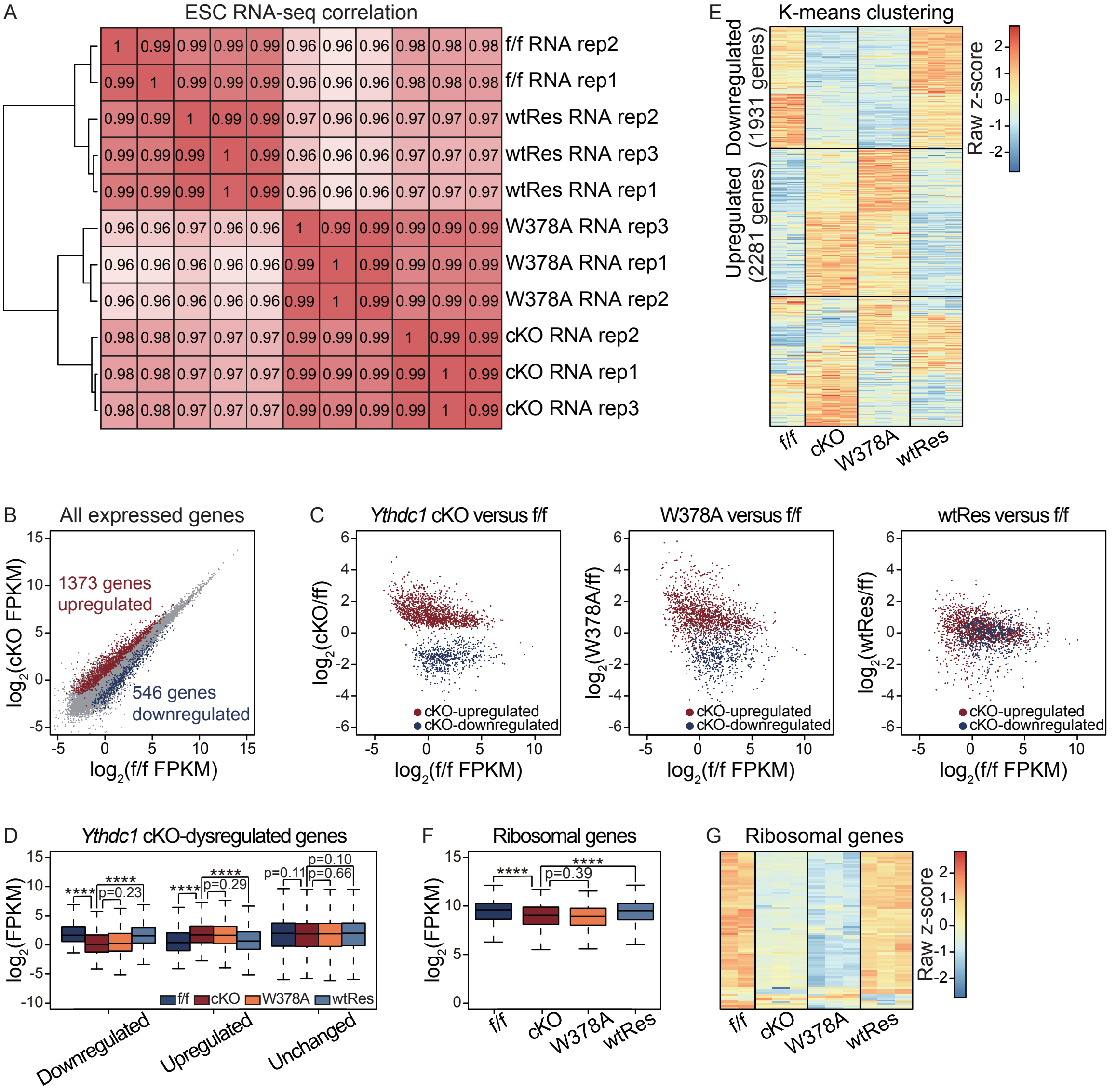

A
ESC RNA-seq correlation
1 0.99 0.99 0.99 0.99 0.96 0.96 0.96 0.98 0.98 0.98 f/f RNA rep2
0.99 1 0.99 0.99 0.99 0.96 0.96 0.96 0.98 0.98 0.98 f/f RNA rep1
0.99 0.99 1 0.99 0.99 0.97 0.96 0.96 0.97 0.97 0.97 wtRes RNA rep2
0.99 0.99 0.99 1 0.99 0.96 0.96 0.96 0.97 0.97 0.97 wtRes RNA rep3
0.99 0.99 0.99 1 0.99 0.96 0.96 0.96 0.97 0.97 0.97 wtRes RNA rep1
0.96 0.96 0.97 0.96 0.96 1 0.99 0.99 0.99 0.99 0.99 W378A RNA rep3
0.96 0.96 0.96 0.96 0.96 0.99 1 0.99 0.99 0.99 0.99 W378A RNA rep1
0.96 0.96 0.96 0.96 0.96 0.99 1 0.99 0.99 0.99 0.99 W378A RNA rep2
0.98 0.98 0.97 0.97 0.97 0.99 0.99 0.99 1 0.99 0.99 cKO RNA rep2
0.98 0.98 0.97 0.97 0.97 0.99 0.99 0.99 0.99 1 0.99 cKO RNA rep1
0.98 0.98 0.97 0.97 0.97 0.99 0.99 0.99 0.99 1 0.99 cKO RNA rep3
E
K-means clustering
Downregulated (1931 genes)
Upregulated (2281 genes)
Raw z-score
f/f
cKO
W378A
wtRes
B
All expressed genes
1373 genes upregulated
546 genes downregulated
log2(cKO FPKM)
log2(f/f FPKM)
C
Ythdc1 cKO versus f/f
log2(cKO/ff)
W378A versus f/f
log2(W378A/ff)
wtRes versus f/f
log2(wtRes/ff)
cKO-upregulated
cKO-downregulated
log2(f/f FPKM)
D
Ythdc1 cKO-dysregulated genes
log2(FPKM)
****
p=0.23
p=0.29
p=0.11
p=0.10
p=0.66
f/f
cKO
W378A
wtRes
Downregulated
Upregulated
Unchanged
F
Ribosomal genes
log2(FPKM)
****
p=0.39
f/f
cKO
W378A
wtRes
G
Ribosomal genes
Raw z-score
f/f
cKO
W378A
wtRes

Fig. S2. ***Ythdc1*** **Deficiency Leads to Transcriptome Defects in ESCs, Related to Fig. 1**

(A) Hierarchical clustering showing that the transcriptome of *Ythdc1* cKO and W378A ESCs was similar, which was relatively different from that of f/f and wtRes ESCs. Pearson's correlation between each replicate calculated in R is labeled in the plot. rep, replicate.

(B) Scatter plot showing the expression level of all expressed genes in *Ythdc1* f/f (x-axis) and cKO (y-axis) ESCs. DEGs defined in *Ythdc1* cKO ESCs with fold change > 2 (Data S1) are labeled in the plot.

(C and D) Expression level of DEGs in *Ythdc1* cKO ESCs (defined as in (B)) could be corrected by WT YTHDC1 protein but not the W378A mutant protein. Scatter plot showing the expression level of genes in *Ythdc1* f/f ESCs (x-axis) and fold change of the level between indicated ESC lines (y-axis) in (C).

(E) K-means clustering (k = 8; Data S1) identified genes simultaneously dysregulated in *Ythdc1* cKO and W378A ESCs.

(F and G) Ribosomal genes were extensively downregulated in *Ythdc1* cKO and W378A ESCs. A total of 83 *Rpl* and *Rps* genes are included in these plots.

Means of replicates were used to generate the summarized data in (B, C, D and F). Significance was calculated with paired two-tailed Student's t test (**** $p < 0.0001$).

Figure S3

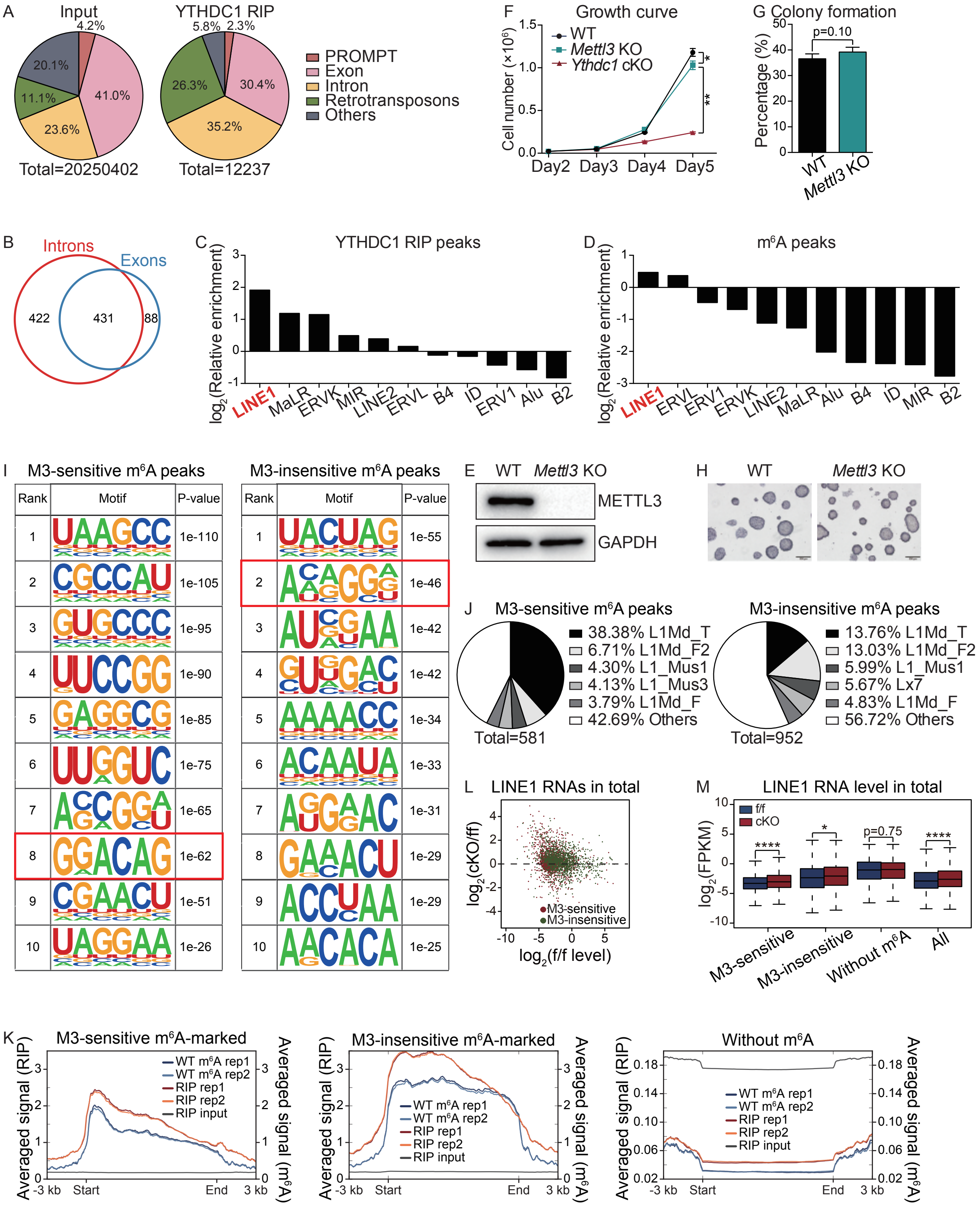
A
Input
YTHDC1 RIP
4.2%
20.1%
11.1%
41.0%
23.6%
Total=20250402
5.8% 2.3%
26.3%
30.4%
35.2%
Total=12237
PROMPT
Exon
Intron
Retrotransposons
Others
F
Growth curve
WT
Mettl3 KO
Ythdc1 cKO
Cell number (×10⁶)
Day2 Day3 Day4 Day5
G Colony formation
p=0.10
Percentage (%)
WT
Mettl3 KO
B
Introns
Exons
422
431
88
C
YTHDC1 RIP peaks
log2(Relative enrichment)
LINE1 MaLR ERVK MIR LINE2 ERVL B4 ID ERV1 Alu B2
D
m6A peaks
log2(Relative enrichment)
LINE1 ERVL ERV1 ERVK LINE2 MaLR Alu B4 ID MIR B2
I
M3-sensitive m6A peaks
Rank Motif P-value
1 UAAGCC 1e-110
2 CGCCAU 1e-105
3 GUGCCC 1e-95
4 UUCCGG 1e-90
5 GAGGCG 1e-85
6 UUGGUC 1e-75
7 ACCGGA 1e-65
8 GGACAG 1e-62
9 CGAACU 1e-51
10 UAGGAA 1e-26
M3-insensitive m6A peaks
Rank Motif P-value
1 UACUAG 1e-55
2 ACAGGA 1e-46
3 AUCGAA 1e-42
4 GUGGAC 1e-42
5 AAAACC 1e-34
6 ACAAUA 1e-33
7 AGGAAC 1e-31
8 GAAACU 1e-29
9 ACCUAA 1e-29
10 AACACA 1e-25
E
WT Mettl3 KO
METTL3
GAPDH
H
WT Mettl3 KO
J
M3-sensitive m6A peaks
38.38% L1Md_T
6.71% L1Md_F2
4.30% L1_Mus1
4.13% L1_Mus3
3.79% L1Md_F
42.69% Others
Total=581
M3-insensitive m6A peaks
13.76% L1Md_T
13.03% L1Md_F2
5.99% L1_Mus1
5.67% Lx7
4.83% L1Md_F
56.72% Others
Total=952
L
LINE1 RNAs in total
log2(cKO/f/f)
log2(f/f level)
M3-sensitive
M3-insensitive
M
LINE1 RNA level in total
f/f
cKO
log2(FPKM)
p=0.75
M3-sensitive
M3-insensitive
Without m6A
All
K
M3-sensitive m6A-marked
M3-insensitive m6A-marked
Without m6A
WT m6A rep1
WT m6A rep2
RIP rep1
RIP rep2
RIP input
Averaged signal (RIP)
Averaged signal (m6A)
-3 kb Start End 3 kb

Fig. S3. RNA Targets of YTHDC1 Revealed by RIP-seq in ESCs, Related to Fig. 2

(A) Pie charts showing the distribution of nuclear RNA input reads (left) and YTHDC1 RIP peaks (right) on different genomic elements in the control ESCs. PROMPT, promoter upstream transcript.

(B) Venn diagram showing the overlap of genes targeted by YTHDC1 at exonic regions (exon targets) and genes targeted by YTHDC1 at intronic regions (intron targets) in the control ESCs.

(C and D) Both YTHDC1 RIP peaks and m^6A peaks were enriched on nuclear LINE1 transcripts in the control ESCs. Representative subfamilies of retrotransposons are included in these graphs.

(E) Western blotting showing that METTL3 protein was eliminated in *Mettl3* KO ESCs.

(F) Growth curve showing that the self-renewal ability was more severely impaired in *Ythdc1* cKO ESCs compared to *Mettl3* KO ESCs. Cell numbers on the last day were used to assess the significance.

(G) Colony formation ability of WT and *Mettl3* KO ESCs was comparable.

(H) AP staining showing that the morphology of colonies was not impacted by *Mettl3* depletion in ESCs.

(I) METTL3-sensitive/insensitive m^6A peaks presented differential motif enrichment patterns revealed by HOMER (related to Fig. 2 J). M3, METTL3.

(J) Pie charts showing the distribution of METTL3-sensitive/insensitive m^6A peaks among LINE1 subfamilies in ESCs, and the top 5 subfamilies with the most m^6A peaks in each group are shown. m^6A peaks with fold enrichment > 6 were considered in this analysis.

(K) Profiles generated by deepTools showing the distribution of YTHDC1 RIP signal and m^6A IP signal on bodies of METTL3-sensitive m^6A-marked (left), METTL3-

insensitive m^6A-marked (middle) and unmarked (right) nuclear LINE1 RNAs in ESCs. M3, METTL3.

(L and M) Scatter plot and boxplot showing the level of LINE1 transcripts in total. Numbers of analyzed LINE1 transcripts are indicated in the legend of Fig. 2 L and 2 M. M3, METTL3.

caRNA m^6A-seq data of WT and *Mettl3*$^{-/-}$-1 ESCs (GSE133600) published by Liu et al. (2020) were used for the analyses in (D, I, J, K, L and M). Data are presented as means with SDs (n = 3 in (F) and n = 4 in (G)). Means of replicates were used to generate the summarized data in (L and M). Significance (* $p < 0.05$, ** $p < 0.01$, **** $p < 0.0001$) was calculated with two-tailed Student's t test (unpaired in (F and G) and paired in (M)).

Figure S4

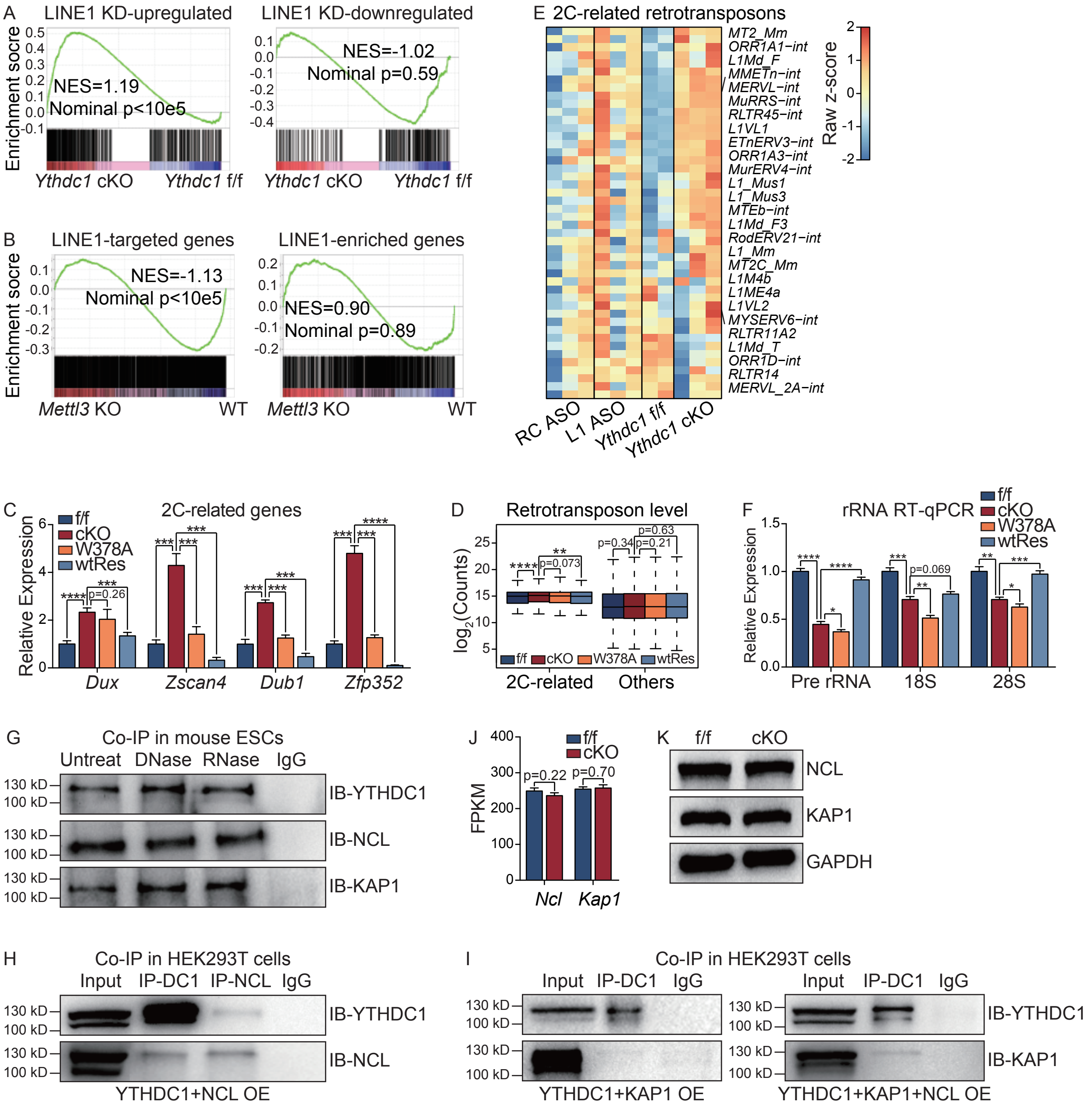
A
LINE1 KD-upregulated
LINE1 KD-downregulated
Enrichment socre
NES=1.19
Nominal p<10e5
NES=-1.02
Nominal p=0.59
Ythdc1 cKO
Ythdc1 f/f
B
LINE1-targeted genes
LINE1-enriched genes
NES=-1.13
Nominal p<10e5
NES=0.90
Nominal p=0.89
Mettl3 KO
WT
E 2C-related retrotransposons
MT2_Mm
ORR1A1-int
L1Md_F
MMETn-int
MERVL-int
MuRRS-int
RLTR45-int
L1VL1
ETnERV3-int
ORR1A3-int
MurERV4-int
L1_Mus1
L1_Mus3
MTEb-int
L1Md_F3
RodERV21-int
L1_Mm
MT2C_Mm
L1M4b
L1ME4a
L1VL2
MYSERV6-int
RLTR11A2
L1Md_T
ORR1D-int
RLTR14
MERVL_2A-int
Raw z-score
RC ASO
L1 ASO
Ythdc1 f/f
Ythdc1 cKO
C
2C-related genes
Relative Expression
f/f
cKO
W378A
wtRes
p=0.26
Dux
Zscan4
Dub1
Zfp352
D
Retrotransposon level
log2(Counts)
p=0.073
p=0.34
p=0.21
p=0.63
2C-related
Others
F
rRNA RT-qPCR
Relative Expression
p=0.069
Pre rRNA
18S
28S
G
Co-IP in mouse ESCs
Untreat
DNase
RNase
IgG
130 kD
100 kD
IB-YTHDC1
IB-NCL
IB-KAP1
J
FPKM
p=0.22
p=0.70
Ncl
Kap1
K
f/f
cKO
NCL
KAP1
GAPDH
H
Co-IP in HEK293T cells
Input
IP-DC1
IP-NCL
IgG
IB-YTHDC1
IB-NCL
YTHDC1+NCL OE
I
Co-IP in HEK293T cells
Input
IP-DC1
IgG
IB-YTHDC1
IB-KAP1
YTHDC1+KAP1 OE
YTHDC1+KAP1+NCL OE

Fig. S4. *Ythdc1* deficient ESCs share transcriptome defects with LINE1 knockdown ESCs, Related to Fig. 3

(A) GSEA showing that LINE1 KD-upregulated genes were also derepressed in *Ythdc1* cKO ESCs. LINE1 KD-dysregulated genes (Percharde et al., 2018) were defined as in Fig. 3 A.

(B) GSEA showing that neither LINE1 RNA-targeted genes nor LINE1 sequence-enriched genes (Lu et al., 2020) were upregulated in *Mettl3* KO ESCs. NES, normalized enrichment score.

(C) RT-qPCR analysis showing that representative 2C-related genes were upregulated in *Ythdc1* cKO ESCs.

(D) 2C-related retrotransposons (Macfarlan et al., 2012; defined as in Fig. 3 G) were upregulated upon *Ythdc1* depletion in ESCs, which could not be rescued by W378A YTHDC1.

(E) Many 2C-related retrotransposons (Macfarlan et al., 2012; defined as in Fig. 3 G) were consistently upregulated in LINE1 KD ESCs and *Ythdc1* cKO ESCs. RC ASO, negative control of LINE1 KD. L1 ASO, LINE1 RNA KD by ASO.

(F) RT-qPCR analysis showing that rRNA level was decreased in *Ythdc1* cKO and W378A ESCs. Primers for pre rRNA target the 5' external transcribed spacer (ETS).

(G) Co-IP in mouse ESCs showing that the interaction between endogenous YTHDC1 and NCL-KAP1 proteins was conserved after the treatment of 0.1 units/μL DNase or 50 μg/mL RNase.

(H and I) Co-IP in HEK293T cells showing the interaction between exogenously expressed mouse YTHDC1 and NCL proteins, and the interaction between exogenously expressed mouse YTHDC1 and KAP1 proteins was more obvious with the ectopic expression of mouse NCL protein. OE, over expression. DC1, YTHDC1.

(J and K) RT-qPCR analysis and western blotting showing that the RNA and protein level of *Ncl* and *Kap1* was comparable in *Ythdc1* f/f and cKO ESCs.

Data are presented as means with SDs ($n = 3$ in (C, F and J)). Means of replicates were used to generate the summarized data in (D). Significance (* $p < 0.05$, ** $p < 0.01$, *** $p < 0.001$, **** $p < 0.0001$) was calculated with two-tailed Student's t test (paired in (D) and unpaired in (C, F and J)).

Figure S5

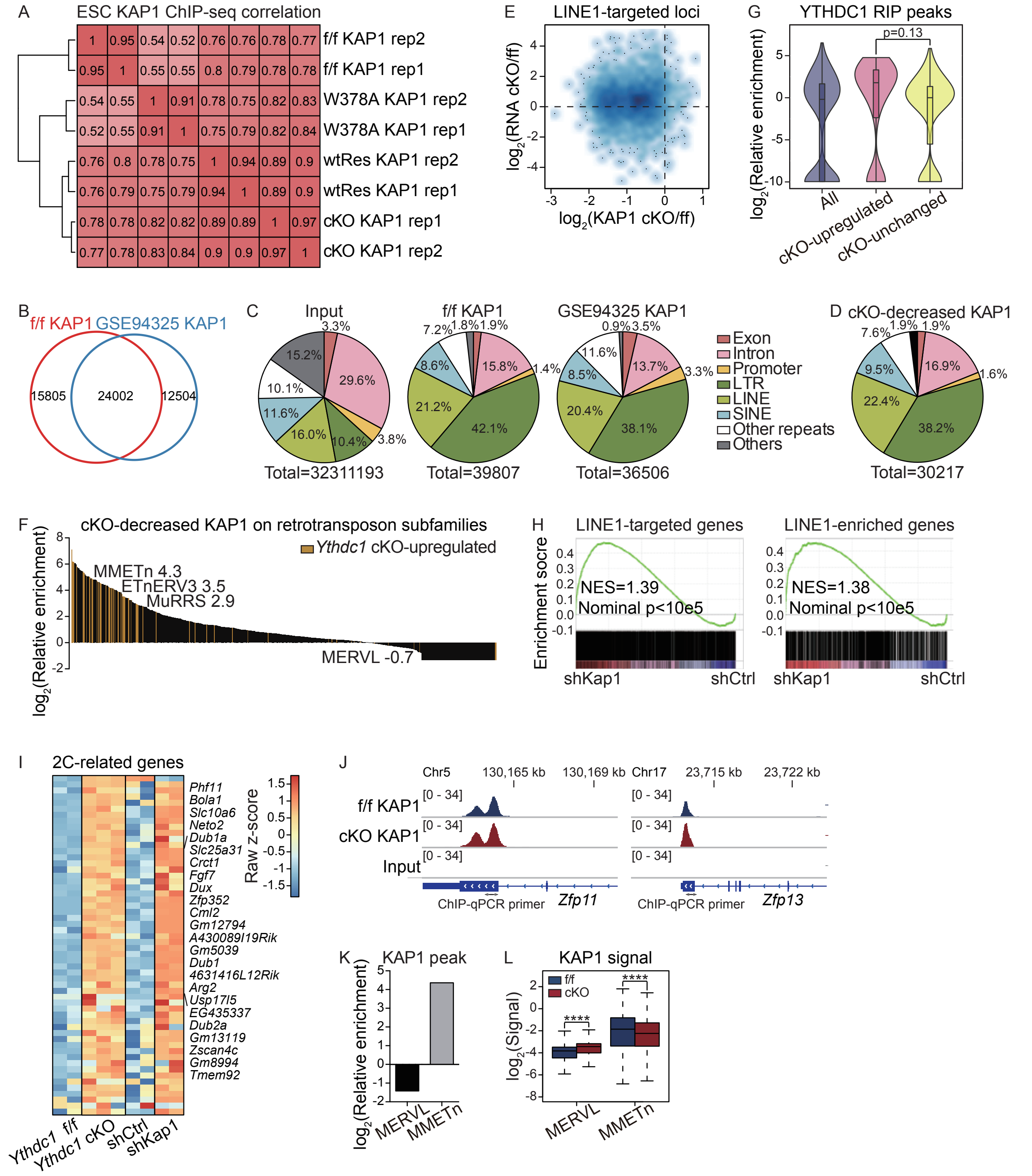

A
ESC KAP1 ChIP-seq correlation
f/f KAP1 rep2
f/f KAP1 rep1
W378A KAP1 rep2
W378A KAP1 rep1
wtRes KAP1 rep2
wtRes KAP1 rep1
cKO KAP1 rep1
cKO KAP1 rep2
B
f/f KAP1
GSE94325 KAP1
15805
24002
12504
C
Input
f/f KAP1
GSE94325 KAP1
Total=32311193
Total=39807
Total=36506
Exon
Intron
Promoter
LTR
LINE
SINE
Other repeats
Others
D
cKO-decreased KAP1
Total=30217
E
LINE1-targeted loci
log2(RNA cKO/ff)
log2(KAP1 cKO/ff)
G
YTHDC1 RIP peaks
p=0.13
log2(Relative enrichment)
All
cKO-upregulated
cKO-unchanged
F
cKO-decreased KAP1 on retrotransposon subfamilies
Ythdc1 cKO-upregulated
MMETn 4.3
ETnERV3 3.5
MuRRS 2.9
MERVL -0.7
log2(Relative enrichment)
H
LINE1-targeted genes
LINE1-enriched genes
Enrichment socre
NES=1.39
Nominal p<10e5
NES=1.38
Nominal p<10e5
shKap1
shCtrl
I
2C-related genes
Phf11
Bola1
Slc10a6
Neto2
Dub1a
Slc25a31
Crct1
Fgf7
Dux
Zfp352
Cml2
Gm12794
A430089I19Rik
Gm5039
Dub1
4631416L12Rik
Arg2
Usp17l5
EG435337
Dub2a
Gm13119
Zscan4c
Gm8994
Tmem92
Raw z-score
Ythdc1 f/f
Ythdc1 cKO
shCtrl
shKap1
J
Chr5
130,165 kb
130,169 kb
Chr17
23,715 kb
23,722 kb
f/f KAP1
cKO KAP1
Input
[0 - 34]
ChIP-qPCR primer
Zfp11
Zfp13
K
KAP1 peak
log2(Relative enrichment)
MERVL
MMETn
L
KAP1 signal
f/f
cKO
****
log2(Signal)
MERVL
MMETn

Fig. S5. Genome Recruitment of KAP1 is Inhibited by *Ythdc1* Depletion in ESCs, Related to Fig. 4

(A) Hierarchical clustering showing the high reproducibility of KAP1 ChIP-seq replicates in each ESC line. Pearson's correlation between each replicate calculated in R is labeled in the plot. rep, replicate.

(B) Venn diagram showing the overlap of KAP1 ChIP peaks defined in *Ythdc1* f/f ESCs in our study and previously identified KAP1 ChIP peaks in control mouse ESCs (De Iaco et al., 2017; GSE94325).

(C) Pie charts showing the distribution of genomic DNA input reads (left), KAP1 ChIP peaks defined in *Ythdc1* f/f ESCs (middle) and previously identified KAP1 ChIP peaks in control mouse ESCs (right; De Iaco et al., 2017; GSE94325) on different genomic elements. LTR, long terminal repeat. SINE, short interspersed nuclear element.

(D) Pie chart showing the genome distribution of *Ythdc1* cKO-decreased KAP1 peaks defined in Fig. 4 A. Color legend of this chart is shared with (C).

(E) Scatter plot showing fold change of KAP1 ChIP signal (x-axis) and RNA level (y-axis) upon *Ythdc1* depletion in ESCs at LINE1 RNA-targeted loci (defined by Lu et al., 2020; related to Fig. 4 D and 4 E).

(F) Many *Ythdc1* cKO-upregulated retrotransposons (defined as in Fig. 3 F) were highly enriched with *Ythdc1* cKO-decreased KAP1 peaks (defined in Fig. 4 A). Retrotransposon subfamilies were ranked in descending order according to the relative peak enrichment. Representative 2C-related retrotransposons are labeled in the plot.

(G) Violin plot showing that the relative enrichment of YTHDC1 RIP peaks on *Ythdc1* cKO-upregulated/unchanged retrotransposons (defined as in Fig. 3 F) was comparable.

(H) GSEA showing the global upregulation of LINE1 RNA-targeted genes (left) and LINE1 sequence-enriched genes (right) in *Kap1* KD ESCs. These genes were defined by Lu et al. (2020). NES, normalized enrichment score.

(I) Many 2C-related genes (Macfarlan et al., 2012; defined as in Fig. 3 C) were consistently upregulated in *Ythdc1* cKO ESCs and *Kap1* KD ESCs. shCtrl, control shRNA expressed. shKap1, *Kap1* shRNA expressed.

(J) IGV tracks showing that the KAP1 occupancy on 3' exon of *Zfp11* (left) and *Zfp13* (right) gene was comparable in *Ythdc1* f/f and cKO ESCs. Normalized BigWig files of replicates are combined for each sample in this view.

(K) KAP1 ChIP peaks defined in *Ythdc1* f/f ESCs were not enriched at MERVL elements but highly enriched at MMETn elements.

(L) KAP1 ChIP signal was not decreased at MERVL elements but significantly decreased at MMETn elements upon *Ythdc1* depletion in ESCs. A total of 2555 MERVL elements along with 1638 MMETn elements were analyzed.

Means of replicates were used to generate the summarized data in (E and L). Significance (**** $p < 0.0001$) was calculated with two-tailed Student's t test (paired in (L) and unpaired in (G)).

Figure S6

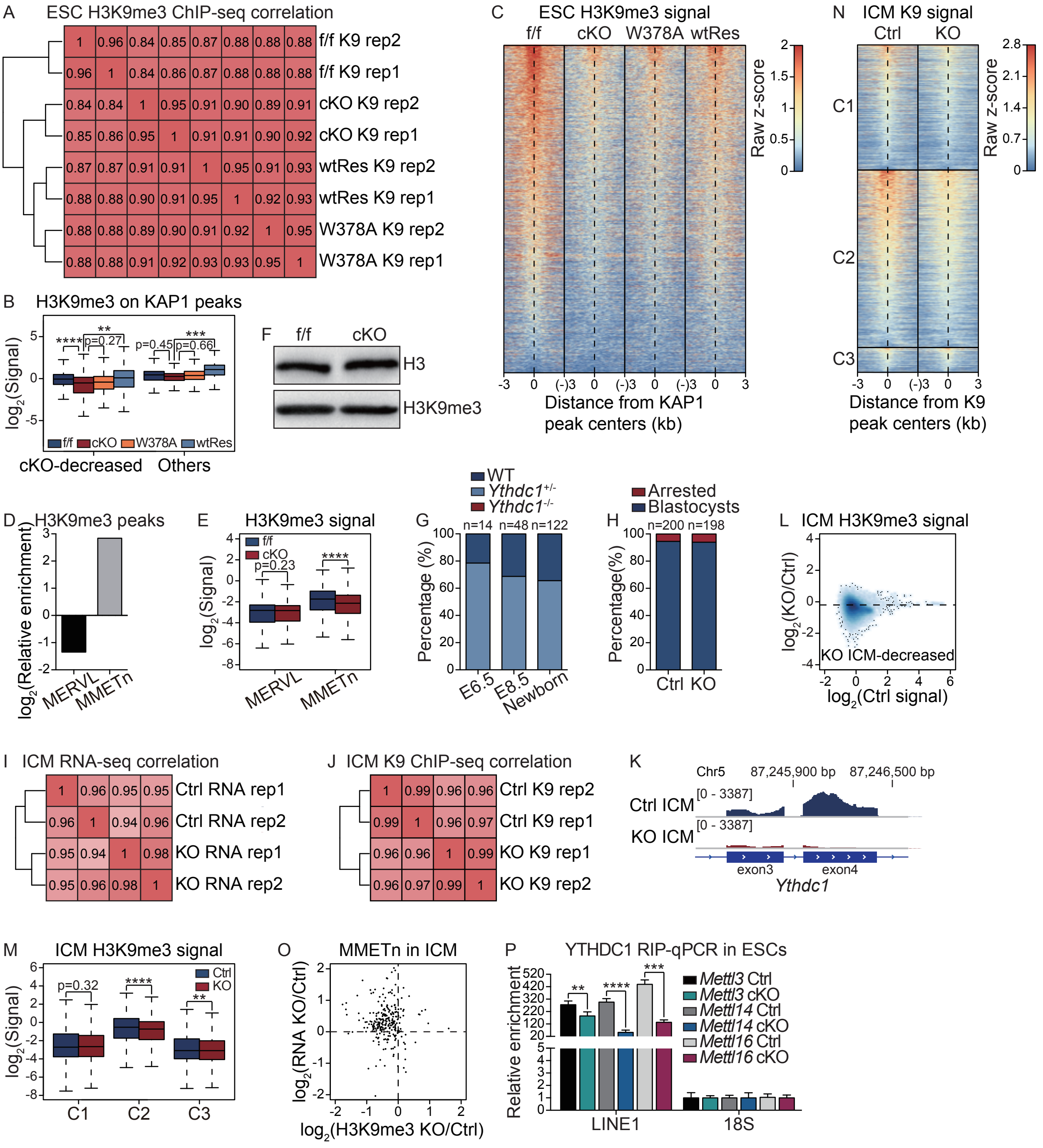

A ESC H3K9me3 ChIP-seq correlation
f/f K9 rep2
f/f K9 rep1
cKO K9 rep2
cKO K9 rep1
wtRes K9 rep2
wtRes K9 rep1
W378A K9 rep2
W378A K9 rep1
B H3K9me3 on KAP1 peaks
log2(Signal)
f/f cKO W378A wtRes
cKO-decreased
Others
C ESC H3K9me3 signal
f/f cKO W378A wtRes
Raw z-score
Distance from KAP1 peak centers (kb)
N ICM K9 signal
Ctrl KO
C1
C2
C3
Raw z-score
Distance from K9 peak centers (kb)
F f/f cKO
H3
H3K9me3
D H3K9me3 peaks
log2(Relative enrichment)
MERVL
MMETn
E H3K9me3 signal
f/f
cKO
p=0.23
log2(Signal)
MERVL
MMETn
G
WT
Ythdc1+/-
Ythdc1-/-
n=14 n=48 n=122
Percentage (%)
E6.5
E8.5
Newborn
H
Arrested
Blastocysts
n=200 n=198
Percentage(%)
Ctrl KO
L ICM H3K9me3 signal
log2(KO/Ctrl)
KO ICM-decreased
log2(Ctrl signal)
I ICM RNA-seq correlation
Ctrl RNA rep1
Ctrl RNA rep2
KO RNA rep1
KO RNA rep2
J ICM K9 ChIP-seq correlation
Ctrl K9 rep2
Ctrl K9 rep1
KO K9 rep1
KO K9 rep2
K
Chr5
87,245,900 bp
87,246,500 bp
Ctrl ICM
[0 - 3387]
KO ICM
[0 - 3387]
exon3
exon4
Ythdc1
M ICM H3K9me3 signal
Ctrl
KO
p=0.32
log2(Signal)
C1
C2
C3
O MMETn in ICM
log2(RNA KO/Ctrl)
log2(H3K9me3 KO/Ctrl)
P YTHDC1 RIP-qPCR in ESCs
Relative enrichment
Mettl3 Ctrl
Mettl3 cKO
Mettl14 Ctrl
Mettl14 cKO
Mettl16 Ctrl
Mettl16 cKO
LINE1
18S

Fig. S6. *Ythdc1* Depletion Disturbs H3K9me3 Deposition on 2C Retrotransposons in Mouse ESCs and Early Embryos, Related to Fig. 5

(A) Hierarchical clustering showing the high reproducibility of H3K9me3 ChIP-seq replicates in each ESC line. Pearson's correlation between each replicate calculated in R is labeled in the plot. rep, replicate. K9, H3K9me3.

(B) H3K9me3 ChIP signal was significantly decreased on *Ythdc1* cKO-decreased KAP1 peaks (defined in Fig. 4 A) in *Ythdc1* cKO and W378A ESCs.

(C) Heatmap showing the H3K9me3 ChIP signal on regions spanning loci of *Ythdc1* cKO-upregulated retrotransposons (defined as in Fig. 3 F) in each ESC line (related to Fig. 5 D). A total of 9706 expressed retrotransposon elements possessing KAP1 peaks are included in the heatmap.

(D) H3K9me3 ChIP peaks defined in *Ythdc1* f/f ESCs were not enriched at MERVL elements but highly enriched at MMETn elements.

(E) H3K9me3 ChIP signal was not decreased on MERVL elements but significantly decreased on MMETn elements upon *Ythdc1* depletion in ESCs. A total of 2555 MERVL elements along with 1638 MMETn elements were analyzed.

(F) Western blotting showing that the H3K9me3 level in total was comparable in *Ythdc1* f/f and cKO ESCs.

(G) *Ythdc1* KO embryos from intercrosses of *Ythdc1*$^{+/-}$ mice could not be detected after E6.5.

(H) Proportion of blastocysts was comparable at E3.5 in Ctrl and *Ythdc1* KO embryos.

(I and J) Hierarchical clustering showing the high reproducibility of RNA-seq and H3K9me3 ChIP-seq replicates in ICM samples. Pearson's correlation between each replicate calculated in R is labeled in the plot. rep, replicate.

(K) IGV tracks showing that *Ythdc1* was successfully depleted by CRISPR/Cas9 technology in mouse embryos. The RNA-seq read coverage on exons 3-4 of *Ythdc1* gene in ICM samples is displayed. Normalized BigWig files of replicates are combined for each sample in this view.

(L) Scatter plot showing the H3K9me3 ChIP signal in Ctrl ICM (x-axis) and fold change of the signal upon *Ythdc1* depletion in ICM (y-axis) on 31553 H3K9me3 peaks identified in Ctrl ICM. *Ythdc1* A total of 21424 KO ICM-decreased H3K9me3 peaks were defined as H3K9me3 peaks with $\log_2$(fold change) < -0.2.

(M and N) H3K9me3 ChIP signal was significantly decreased on group C2 retrotransposons (defined in Fig. 5 J) in *Ythdc1* KO ICM. A total of 43541, 17572 and 13595 expressed elements of group C1, C2 and C3 retrotransposons were analyzed in (M), respectively, and a total of 7373, 10466 and 1460 elements possessing H3K9me3 peaks are included in the heatmap in (N). K9, H3K9me3.

(O) Scatter plot showing fold change of the H3K9me3 ChIP signal (x-axis) and RNA level (y-axis) upon *Ythdc1* depletion in ICM on genome loci of MMETn elements. A total of 263 expressed MMETn elements are included in this plot.

(P) YTHDC1 RIP-qPCR analysis showing that the association of YTHDC1 with nuclear LINE1 RNAs was attenuated in *Mettl3*/*Mettl14*/*Mettl16* cKO ESCs. Relative enrichment was calculated as the percent of input relative to the negative control antibody IgG. Two independent reactions for each IP were performed. Data are presented as means with SDs (n = 3 technical replicates).

Means of replicates were used to generate the summarized data in (B, C, E, L, M, N and O). Significance (* $p < 0.05$, ** $p < 0.01$, *** $p < 0.001$, **** $p < 0.0001$) was calculated with two-tailed Student's t test (paired in (B, E and M) and unpaired in (P)).
